# Unraveling the pleiotropic roles of the mTOR-glycolytic axis in ulcerative colitis: from immunometabolic dysregulation to mucosal barrier remodeling

**DOI:** 10.3389/fimmu.2026.1889686

**Published:** 2026-09-02

**Authors:** Yanjie Chen, JinYin Xiao, Limin Xiao, Anni Chen, Lizhi He, Zhenquan Wang

**Affiliations:** 1 Department of Anorectal, the Second Affiliated Hospital of Hunan University of Traditional Chinese Medicine, Changsha, China; 2 Graduate School of Hunan University of Chinese Medicine, Changsha, China

**Keywords:** glycolysis, immune metabolism, mTOR signaling pathway, mTOR-glycolytic axis, ulcerative colitis

## Abstract

Ulcerative colitis (UC) is a chronic, relapsing inflammatory disorder of the colonic mucosa, whose pathogenesis is intricately linked to metabolic reprogramming within both immune and epithelial compartments. The mechanistic target of rapamycin (mTOR) signaling pathway serves as a central immunometabolic hub that integrates nutrient availability, microbial cues, and inflammatory signals to orchestrate glycolytic flux, thereby profoundly shaping the functional plasticity of diverse intestinal cell populations. This review systematically delineates, from a cell-type-specific perspective, the divergent regulatory roles of the mTOR-glycolysis axis in intestinal immunity and mucosal barrier homeostasis. We first outline the core molecular architecture of mTORC1/mTORC2-driven glycolytic reprogramming, highlighting key regulatory nodes including GLUT1/3-mediated glucose uptake, HK2-dependent rate-limiting phosphorylation, and PKM2-governed metabolic-transcriptional switching. Subsequently, we examine how aberrant mTOR-glycolysis axis activation in neutrophils, macrophages, type 3 innate lymphoid cells, and CD4+ T effector subsets propagates a feed-forward inflammatory loop—exacerbating oxidative burst, NETosis, M1 polarization, and Th17 pathogenicity—while simultaneously undermining the metabolic fitness and suppressive integrity of regulatory T cells. Moreover, we discuss the metabolic rewiring of intestinal epithelial cells via the mTOR-glycolysis axis, which compromises barrier integrity, disrupts epithelial regeneration, and initiates a “metabolic-secretory” crosstalk that perpetuates mucosal inflammation. Collectively, this review positions the mTOR-glycolysis axis as a rheostat governing the transition from homeostatic immunosurveillance to pathogenic inflammation in UC, and proposes that cell-selective metabolic checkpoint targeting—rather than broad systemic inhibition—represents a promising precision strategy for future therapeutic intervention.

## 1 Introduction

Ulcerative colitis (UC) is a chronic, idiopathic inflammatory bowel disease (IBD) characterized by a relapsing-remitting course of colonic mucosal inflammation. Typically, the disease process originates in the rectum and propagates proximally in a continuous fashion, involving variable extents of the colon (1). Although the incidence of UC has historically predominated in Western nations, its global epidemiological landscape is undergoing a profound transition. While trends in high-income countries have begun to plateau, newly industrialized regions—including Asia, Latin America, and Africa—are witnessing a rapid and disproportionate surge in disease prevalence (2). The pathogenesis of UC is widely conceptualized as a multifactorial process wherein environmental triggers and dysregulated microbial metabolic signatures converge in genetically susceptible individuals to incite a chronic, aberrant immune response against the colonic mucosa, ultimately culminating in the collapse of epithelial barrier homeostasis (3). To date, the triad of intestinal epithelial barrier integrity, host immune reactivity, and gut microbial metabolic homeostasis remains pivotal in both the pathogenesis and therapeutic landscape of UC. The management paradigm has undergone a fundamental shift from reactive symptom control toward proactive, target-driven strategies centered on “mucosal healing”. Nevertheless, conventional pharmacological interventions—including aminosalicylates, corticosteroids, immunomodulators, and biologics—encounter a formidable “therapeutic ceiling,” with a significant subset of patients exhibiting primary non-response or secondary loss of response to existing biologics and small-molecule inhibitors (4). Therefore, elucidating UC pathogenesis from novel mechanistic perspectives and identifying innovative therapeutic targets are of paramount importance for overcoming the limitations inherent in current clinical interventions.

mTOR is a highly conserved serine/threonine kinase that acts as a pivotal immunometabolic hub. It integrates extrinsic signals, including nutrient availability and immune challenges, to coordinate essential cellular processes such as growth, autophagy, and immune cell lineage specification (5). Emerging evidence indicates that the mTOR signaling pathway integrates luminal nutrient cues with host immune inputs to modulate the reciprocal balance between pro-inflammatory T helper 17 (Th17) and regulatory T (Treg) cells. Dysregulation of this signaling axis precipitates the breakdown of intestinal immune tolerance and exacerbates the chronic inflammatory milieu characteristic of UC (6). By positioning the mTOR pathway at the nexus of luminal metabolic equilibrium and systemic immune reactivity, fine-tuning this signaling axis offers a dual therapeutic potential: not only to dampen refractory inflammation but also to reinstate metabolic homeostasis within the intestinal epithelial cells of UC patients (7, 8). Beyond its canonical role in bioenergetic replenishment, glycolysis serves as a critical metabolic nexus that shunts glucose-derived carbon intermediates into divergent biosynthetic branches. This redirection fulfills the heightened anabolic demands of cells and fuels the inflammatory effector functions of activated immune subsets (9). Evidence suggests that this metabolic transition toward accelerated glycolysis—commonly termed metabolic reprogramming—functions as a fundamental bioenergetic engine. It governs the pro-inflammatory polarization of mucosal macrophages and the differentiation of Th17 cells, thereby precipitating a cytokine storm and exacerbating the breakdown of intestinal immune tolerance (10). Consequently, the aberrant upregulation of glycolysis within the intestinal microenvironment represents a pivotal pathophysiological driver that transcends mere bioenergetic provision. During UC pathogenesis, the mTOR signaling pathway functions as a central immunometabolic hub, orchestrating glycolytic reprogramming across diverse intestinal cell populations and profoundly impacting both mucosal barrier integrity and intestinal immunity (11). Accordingly, this review adopts a cell-type-specific framework to systematically delineate the divergent regulation of the mTOR-glycolysis axis in intestinal immunity and barrier integrity, aiming to provide novel strategic perspectives and therapeutic avenues for the management of UC.

## 2 Overview of the core molecular mechanisms governing the mTOR-glycolysis axis

### 2.1 Molecular mechanisms of synergistic metabolic reprogramming driven by mTORC1 and mTORC2 complexes

mTOR functions through two distinct complexes: mTORC1 and mTORC2. As a central metabolic sensor, mTORC1 integrates diverse cues—including amino acids, growth factors, energy status (ATP/AMP), and oxygen levels—via its precise lysosomal localization. In contrast, mTORC2 primarily responds to growth factor signaling and associates with functional ribosomes to integrate the cellular translational state (12, 13). Mechanistically, mTORC1 synergizes with the core transcriptional networks of HIF-1α and c-Myc to remodel the glycolytic landscape (14). The mTORC1-eIF4E axis not only stimulates the translation of HIF-1α mRNA but also counteracts its VHL-mediated degradation under normoxia through robust protein synthesis. This culminates in the pathological stabilization of HIF-1α protein, thereby driving the transcription of GLUT1, HK2, PFKFB3, and LDHA (15). Simultaneously, mTORC1 enhances both the translation and protein stability of c-Myc by inhibiting its ubiquitination-mediated degradation. In turn, c-Myc directly drives the transcription of virtually all glycolytic enzymes and PDK1, compelling the cells into a lactate-producing mode (16). Through this HIF-1α/c-Myc transcriptional axis, mTORC1 establishes a positive feedback loop that sustains high glycolytic flux, thereby meeting the biosynthetic demands of rapidly proliferating cells (17, 18). mTORC2 modulates glycolytic metabolism through various downstream effector pathways, with the AKT signaling axis serving as the primary regulator. Upon growth factor stimulation, mTORC2 phosphorylates AKT at Ser473; subsequently, activated AKT drives the glycolytic program at both transcriptional and post-translational levels (12). At the transcriptional level, AKT promotes the nuclear translocation of SREBP1c and upregulates glucokinase (GCK) expression, thereby facilitating glucose phosphorylation and channeling glucose-derived carbons into the glycolytic pathway. Post-translationally, mTORC2-activated AKT modulates the activity of GSK3β, whose inactivation relieves the inhibition of multiple downstream metabolic targets (19). In the liver, the mTORC2-AKT-glucokinase-SREBP1c axis orchestrates the coordination of glucose uptake and glycolytic pathways with *de novo* lipogenesis, thereby coupling carbohydrate catabolism to systemic metabolic homeostasis (20). In the context of cancer, the mTORC2-AKT signaling pathway interacts with PFKFB2, a rate-limiting regulator of glycolysis. PFKFB2 functions by promoting the production of fructose-2,6-bisphosphate, thereby sustaining high glycolytic rates in proliferating cells (21, 22).

### 2.2 Key regulatory nodes of glycolysis

Intestinal metabolic reprogramming relies on the exquisite modulation of multiple key nodes within the glycolytic pathway (**Table 1**). Specifically, glucose uptake, HK2-catalyzed rate-limiting phosphorylation, and the dual metabolic and transcriptional roles of PKM2 constitute the core regulatory framework of glycolysis.

**Table 1:** Clinical biomarkers related to glycolytic metabolism in UC.

Biomarker	Detection of source	The role in glycolysis metabolism	Correlation with UC clinical manifestations	References
PKM2	Peripheral serum	PKM2 is the rate-limiting enzyme for glycolysis. Under inflammatory conditions, PKM2 transforms from a tetramer to a dimer, thereby inducing the release of pro-inflammatory factors.	It is positively correlated with the Mayo score and CDAI score of UC, as well as the endoscopic activity. It is also significantly correlated with CRP and ESR.	(23)
lactic acid	faeces	Lactic acid is the final product of glycolysis. Accumulation of lactic acid indicates a shift in metabolism towards aerobic glycolysis, leading to acidosis in tissues.	The fecal lactate levels in patients with active ulcerative colitis are significantly elevated. Excessive accumulation of lactate can lead to a decrease in intestinal pH, exacerbate mucosal damage, and indicate a lower clinical remission rate.	(24)
HIF-1α	Intestinal mucosa Biopsy	The upstream regulators and downstream targets of PKM2. It drives the transcription of glycolytic genes such as GLUT1 and LDHA.	Epithelial cells express HIF-1α, which promotes repair, while immune cells express HIF-1α, which mediates damage. This is the core for evaluating the “spatiotemporal metabolic landscape”.	(25)
PDK2	Intestinal mucosa Biopsy	The increase in PDK2 indicates a complete shift of the metabolic flow from the mitochondrial cycle to glycolysis, and it is a driver of tissue inflammation and oxidative stress.	PDK2 is significantly upregulated in patients with active ulcerative colitis. High expression indicates severe mitochondrial dysfunction and a lower rate of clinical remission.	(26)
GLUT1	Intestinal mucosa Biopsy	GLUT1 is the core transporter for cells to take in glucose. Its high expression directly drives a large influx of glucose, providing sufficient substrates for the subsequent glycolysis process.	The expression intensity and spatial distribution of GLUT1 reflect the metabolic requirements of the tissue. In UC, it is a clear biomarker of high metabolic activity of cells.	(27)
HK2	Intestinal mucosa Biopsy	HK2 efficiently utilizes ATP to phosphorylate glucose into 6-phosphoglucose by binding to the outer mitochondrial membrane, thereby irreversibly initiating and maintaining a high glucose glycolysis flux.	The expression of HK2 at the top indicates that the epithelial cells are undergoing severe metabolic remodeling, suggesting that the mucosal barrier is about to develop ulcers or erosions. It can be used as an early biochemical warning indicator before endoscopic examination.	(28)

#### 2.2.1 Glucose uptake: GLUT1/3-mediated regulation of the metabolic gateway

The initiation of glycolysis is predicated on transmembrane glucose transport as a rate-limiting prerequisite, which is primarily mediated by the transporters GLUT1 and GLUT3. Research indicates that while GLUT1 is ubiquitously overexpressed across various malignancies to sustain basal glucose requirements, GLUT3—possessing a five-fold higher substrate affinity and superior transport velocity—affords cancer cells a competitive advantage in glucose acquisition within low-glucose microenvironments (29). Accordingly, dysregulated GLUT1 and GLUT3 expression is considered a major contributor to the malignant evolution of tumors (30). Acting as a “metabolic switch”, the upregulation of GLUT1/3 facilitates accelerated glycolytic flux in UC mucosa, echoing its established role in cancer biology (31). Mechanistically, the transcriptional landscape is dominated by HIF-1α and c-Myc; stabilized HIF-1α under hypoxic conditions directly activates the transcription of these transporters, whereas c-Myc potently augments their levels via synergistic recruitment to the promoter elements (32). Furthermore, the PI3K/Akt signaling pathway orchestrates multi-layered glycolytic reprogramming by facilitating the membrane translocation and activation of GLUT1, while simultaneously phosphorylating a series of downstream metabolic enzymes, including HK2, PFKFB3/4, and PKM2 (33).

#### 2.2.2 HK2: a mitochondrial-bound regulatory node governing rate-limiting phosphorylation

Upon cellular entry, glucose is phosphorylated into glucose-6-phosphate (G6P) by hexokinase 2 (HK2), a reaction that constitutes the first irreversible and rate-limiting step of glycolysis (34). Distinct from other hexokinase isoforms, HK2 is markedly upregulated in tumor cells and possesses an exclusive mitochondrial-tethering capacity. This localization is orchestrated by its N-terminal hydrophobic domain, which mediates the interaction with the voltage-dependent anion channel (VDAC), thereby anchoring HK2 securely to the outer mitochondrial membrane (35). This subcellular compartmentalization endows HK2 with a dual functional edge: it facilitates the privileged access to mitochondrially exported ATP for glucose phosphorylation, thereby markedly accelerating glycolytic flux and fueling anabolism with abundant biosynthetic precursors. Concurrently, by safeguarding the permeability barrier of the outer mitochondrial membrane, HK2 suppresses cytochrome c release, directly antagonizing the intrinsic apoptotic signaling pathway (36). The transcriptional landscape of HK2 is orchestrated by a network of oncogenic cascades, including the PI3K/Akt (37), MAPK (38), and NF-κB (39) pathways. Beyond its canonical enzymatic hallmarks, emerging evidence identifies HK2 as a signaling transducer that modulates mTORC1 activity and drives the remodeling of the immune microenvironment (40). The mitochondrial dissociation of hexokinase 2 (HK2) represents a pivotal event in the immune cell response to acute stress. Under steady-state conditions or in the presence of pro-survival signaling, Akt suppresses GSK3β activity to maintain the mitochondrial localization of HK2 (41). Conversely, under inflammatory stress, activated GSK3β directly phosphorylates HK2 at residues Thr473 and Ser465, triggering its detachment from the mitochondria. This translocation not only impairs efficient energy coupling but also serves as a critical molecular switch orchestrating neutrophil apoptosis and the oxidative burst (42).

#### 2.2.3 PKM2: a dual-function metabolic and transcriptional hub governed by oligomeric state switching

The terminal regulatory gatekeeper of glycolysis is presided over by Pyruvate Kinase M2 (PKM2), which is distinguished by its ability to execute dual metabolic and transcriptional programs through reversible oligomeric switching. In its highly active tetrameric state, PKM2 catalyzes the irreversible conversion of phosphoenolpyruvate (PEP) to pyruvate, propelling glycolytic carbon flux toward its terminal stage for efficient ATP production. Conversely, when PKM2 dissociates into low-activity dimers, upstream glycolytic intermediates accumulate and are shunted into the pentose phosphate pathway and serine synthesis pathway. This metabolic rewiring provides essential biosynthetic building blocks—including nucleotides, amino acids, and lipids—to sustain the biomass demands of rapidly proliferating tumor cells (43). More pivotally, the dimeric form of PKM2 undergoes nuclear translocation, where it assumes non-canonical roles as both a transcriptional coactivator and a protein kinase (44). In the UC intestinal mucosal inflammatory microenvironment, the equilibrium between PKM2 tetramers and dimers is exquisitely regulated by a diverse repertoire of post-translational modifications (PTMs), FGFR1-mediated Tyr105 phosphorylation (45), Lys305 acetylation (46), and ROS-induced Cys358 oxidation collectively inhibit PKM2 activity (47), thereby diverting metabolic flux toward biosynthetic pathways. Concurrently, pro-inflammatory signaling triggers LDHA phosphorylation at the Tyr10 residue. This modification enhances the binding affinity of LDHA for its substrate NADH, accelerating the conversion of pyruvate to lactate to sustain the rapid energetic demands of immune cells under stress (48). Beyond rapid regulatory mechanisms such as phosphorylation and oxidation, the control of protein stability for key metabolic enzymes is a cornerstone of immune cell metabolic reprogramming. PFKFB3, a master regulator of glycolysis, is highly expressed during immune cell activation to drive ATP production. However, its intracellular abundance is stringently governed by the ubiquitin ligase complex APC/C-Cdh1, which mediates PFKFB3 ubiquitination and subsequent proteasomal degradation, effectively constraining glycolytic flux (49). In the intestinal inflammatory microenvironment, dysregulation of this degradation mechanism can lead to persistent metabolic hyperactivation in neutrophils or macrophages, ultimately exacerbating oxidative stress and tissue damage.

### 2.3 spatiotemporal orchestration of immunometabolism: reshaping immune cell fate decisions via the mTOR-glycolysis axis

While evolutionarily linked by a common catalytic subunit, mTORC1 and mTORC2 function as non-redundant modulators of glycolytic flux within the immune system. This divergence in function is rooted in their differential recruitment of upstream cues and effector substrates, ultimately leading to lineage-specific physiological outcomes (50). mTORC1 functions as a nutrient-sensitive initiator that orchestrates effector-associated glycolytic programs. In CD8+ cytotoxic T lymphocytes (CTLs) and T helper 17 (Th17) cells, TCR engagement alongside co-stimulation triggers mTORC1 activation, which selectively enhances the transcription of HIF-1α mRNA via the S6K1/4E-BP1 signaling axis (51). The accumulated HIF-1α protein complexes with c-Myc to co-activate the glucose transporter GLUT1 and rate-limiting glycolytic enzymes, thereby establishing a high glycolytic flux that is indispensable for clonal expansion and the production of effector cytokines (52). Conversely, in regulatory T cells (Tregs), the inhibition of mTORC1 activity is indispensable for maintaining Foxp3 stability. Unrestrained mTORC1-driven glycolysis compromises their suppressive capacity, underscoring the pivotal role of mTORC1-mediated metabolic regulation as a central arbiter in the fate decisions between effector and regulatory lineages (53). In contrast, mTORC2 is largely dispensable for the acute glycolytic burst in effector cells, yet it serves as a distinct requirement for metabolic adaptation during differentiation and memory formation. Genetic ablation of mTORC2 via Rictor deletion in CD8+ T cells fails to perturb effector-phase glycolysis but severely impairs the generation of long-term memory cells. This indicates that the nexus between mTORC2 and metabolic reconfiguration is decoupled from mTORC1-driven glycolysis and is inextricably linked to cell survival and memory potential (54). Alternatively activated (M2) macrophages harbor a signature mTORC2-dependent glycolytic program. In this state, mTORC2 synergizes with the IL-4/STAT6 signaling axis to drive the induction of the transcription factor IRF4, which subsequently transactivates glycolytic genes essential for M2 polarization and tissue-repair functions (55).

**Figure 1:**
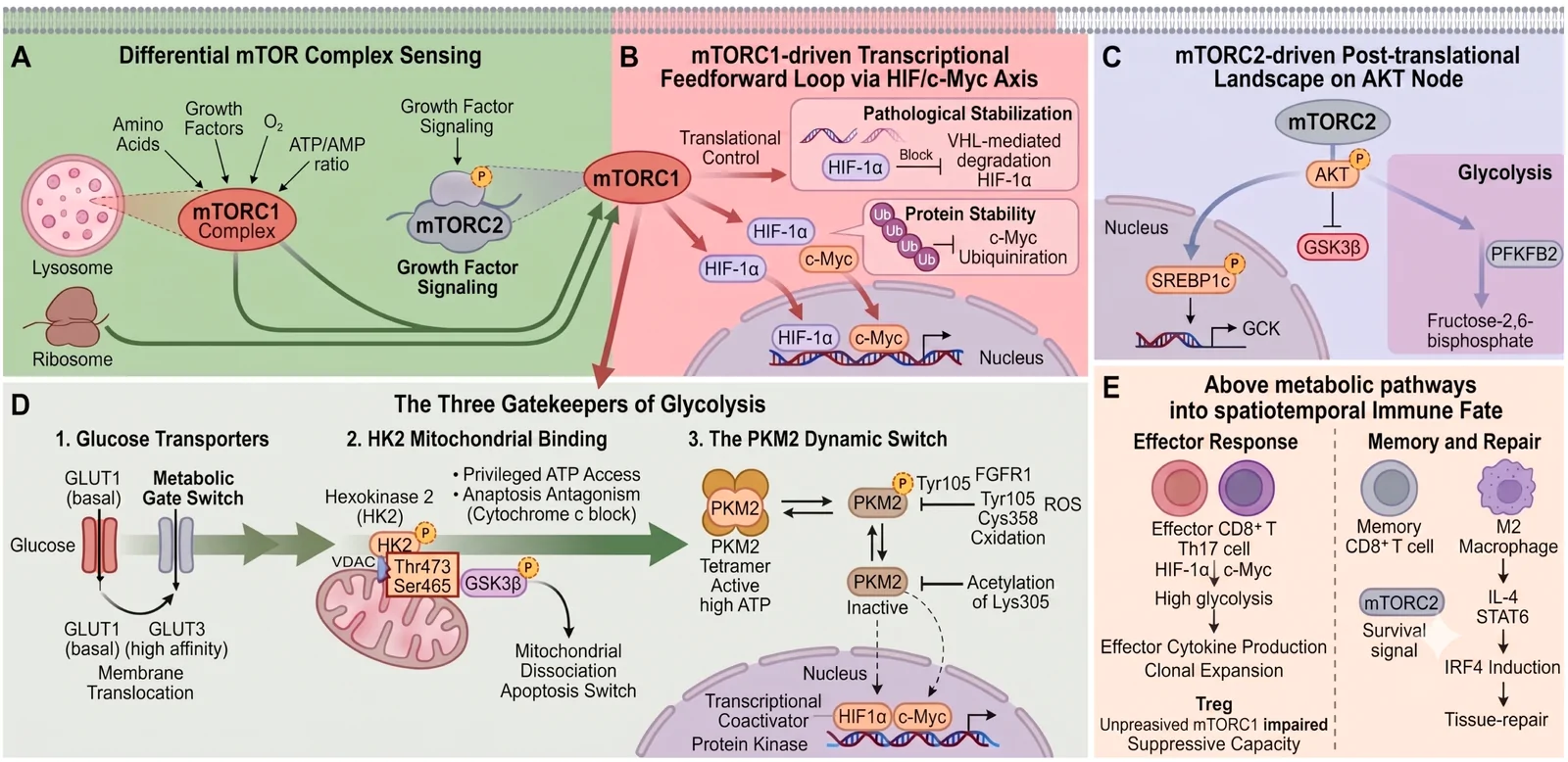
The different roles of mTOR1 and mTOR2 in regulating glycolysis-driven processes. **(A)** Differential mTOR Complex Sensing. **(B)** mTORC1-driven Transcriptional Feed forward Loop via HIF/c-Myc Axis. **(C)** mTORC2-driven Post-translational Landscape on AKT Node. **(D)** The Three Gatekeepers of Glycolysis. **(E)** Above metabolic pathways into spatiotemporal Immune Fate. (mTORC1, mammalian target of rapamycin; PKM2, pyruvate kinase M2 type; GLUT1/3, glucose transporter 1/3; HIF-1α, hypoxia-inducible factor-1 α; AKT, protein kinase B; SREBP1c, sterol regulatory element binding protein 1c; GSK3 β, glycogen synthase kinase 3 β; c-Myc,Myelocytomatosis viral oncogene homolog; IRF4, Interferon Regulatory Factor 4; HK2, Hexokinase 2; PFKFB2, 6-phosphofructo-2-kinase/fructose-2,6-biphosphatase 2).

Consequently, mTORC1 functions as a master switch, coupling nutrient sensing to rapid pro-inflammatory glycolysis; in contrast, mTORC2 serves as a context-dependent regulator that fine-tunes glycolytic programs in alignment with the specific bioenergetic and biosynthetic demands of the immune response, thereby meeting the specialized requirements of memory, regulatory, and homeostatic immunity (56) (**Figure 1**).

## 3 Metabolic dysregulation of the mTOR-glycolysis axis in immune cells: a driver of inflammation

### 3.1 Neutrophils

As the premier rapid-response effectors of the innate immune system, neutrophils undergo massive transepithelial migration to counter microbial invasion within the gut. However, their unbridled activation and the release of a pro-inflammatory secretome often pivot from protective surveillance to become a primary catalyst for mucosal collateral damage (57, 58). Loss of Farnesoid X receptor (FXR) function in neutrophils establishes a pathological feed-forward loop, wherein unrestrained mTORC1 signaling drives metabolic reprogramming toward glycolysis. This heightened glycolytic flux directly fuels a suite of pro-inflammatory effector functions—including excessive reactive oxygen species (ROS) production, neutrophil extracellular trap (NET) formation, and augmented chemotaxis—which collectively exacerbate intestinal mucosal injury (59, 60). Recent studies demonstrate that pharmacological intervention with INT-747 (obeticholic acid), a semi-synthetic bile acid-derived FXR agonist, markedly suppresses mTORC1-mediated glycolytic flux and dampens inflammatory effector responses in neutrophils derived from IBD patients. This modulation effectively re-establishes neutrophil homeostasis and alleviates intestinal inflammation (61). In the pathogenesis of UC, the intestinal inflammatory microenvironment drives neutrophil metabolic reprogramming by activating the mTOR/HIF-1α signaling pathway (62, 63), orchestrating a metabolic shift from oxidative phosphorylation toward a PFKFB3-mediated, high-flux glycolytic state (64). This immunometabolic dysregulation not only significantly enhances the ROS burst and the release of NETs but also perpetuates mucosal barrier injury and inflammatory infiltration through the sustained activation of the ‘mTOR-glycolysis-NETs’ axis (65). Collectively, these findings identify the mTOR-glycolysis axis as a pivotal metabolic switch governing neutrophil pro-inflammatory activation in UC.

### 3.2 Macrophages

As indispensable gatekeepers of mucosal homeostasis, intestinal macrophages leverage their profound functional plasticity to orchestrate an exquisite equilibrium between immunological tolerance toward the commensal microbiota and vigilant immunosurveillance against enteric pathogens (66, 67). The mTOR signaling axis functions as a pivotal metabolic rheostat within macrophages, wherein mTORC1 and mTORC2 orchestrate distinct yet interconnected bioenergetic programs (68) (69). mTORC1 serves as a primary engine driving glycolytic flux by enhancing the translation of HIF-1α and c-Myc; conversely, mTORC2 functions as a metabolic gatekeeper of glucose utilization, modulating Akt-dependent glucose uptake and channeling metabolic intermediates toward either biosynthetic or oxidative pathways (70). This “mTOR-centered metabolic switch” ultimately dictates the functional plasticity of macrophages, bridging the fundamental gap between nutrient sensing and immune polarization (71). Emerging evidence indicates that Dioscin modulates the mTORC1/HIF-1α and mTORC2/PPAR-γ signaling axes to suppress aerobic glycolysis while augmenting fatty acid oxidation, thereby driving M2 macrophage polarization to alleviate intestinal inflammation in UC (72). Furthermore, ZNF667 acts as a negative regulator of the aerobic glycolytic switch; by thwarting mTOR-mediated metabolic reprogramming, it dampens the LPS-induced pro-inflammatory burst in macrophages, effectively depriving bioenergetically demanding inflammatory responses of their fuel and restoring metabolic homeostasis (73). Within the intricate intestinal microenvironment of UC, macrophages integrate signals from hypoxia, pathogen-associated molecular patterns (PAMPs), and pro-inflammatory cytokines to trigger the hyperactivation of the mTORC1 signaling pathway. This culminates in a pathological metabolic rewiring toward aerobic glycolysis, characterized by the stabilization of HIF-1α and the upregulation of key rate-limiting enzymes, including PKM2 and PFKFB3 (63, 74). This shift skews macrophages toward a pathogenic M1 phenotype, leading to the profuse secretion of IL-1βand IL-6 (75). Furthermore, the accumulation of glycolytic byproducts, notably lactate, in the extracellular milieu establishes a feed-forward loop that reinforces mTOR activity and suppresses anti-inflammatory regulators (72, 76). Such mTOR-glycolysis axis-driven immunometabolic disequilibrium not only disrupts macrophage homeostatic maintenance but also represents a core molecular mechanism fueling the chronicity of intestinal inflammation and persistent mucosal barrier impairment in UC. Taken together, mTOR-driven aerobic glycolysis acts as a master immunometabolic regulator governing macrophage phenotypic plasticity. Strategic manipulation of this metabolic switch allows for the redirection of macrophage polarization, thus intercepting diverse pathological progressions ranging from hyper-inflammatory cascades to the maturation of the tumor microenvironment.

### 3.3 Type 3 innate lymphocytes

As quintessential homeostatic sentinels at the intestinal interface, group 3 innate lymphoid cells (ILC3s) integrate microbiota-derived metabolites and neuroendocrine cues via the RORγt-dependent IL-22/IL-17 cytokine axis, thereby precisely orchestrating mucosal barrier integrity and antimicrobial defense (77, 78). Regarding the clinical progression of UC, Gregory F. Sonnenberg posits that the impaired capacity of ILC3s to orchestrate IL-22/IL-23-driven epithelial regenerative programs directly precipitates the collapse of mucosal reparative potential and the perpetuation of colonic tissue injury (79). Intriguingly, this perspective stands in stark contrast to the earlier findings of Lauren A. Zenewicz and more recent evidence from Nicolas Serafini, both of whom concluded that ILC3s confer protection against inflammatory bowel disease in murine models (80, 81). In the context of the mTOR-glycolysis axis in ILC3s, Surace et al. elucidated that ILC3 effector potency is fundamentally anchored in an mTOR-responsive metabolic circuit. This circuit synchronizes accelerated glycolysis with mitochondria-derived redox signaling, thereby furnishing the indispensable bioenergetics and regulatory cues required for the RORγt-driven orchestration of mucosal cytokines (82). Furthermore, research by Sepahi et al. demonstrated that gut microbiota-derived metabolites, such as short-chain fatty acids (SCFAs), calibrate the metabolic thresholds of ILC3s via G protein-coupled receptors (GPCRs) and the mTOR signaling pathway (83). Collectively, the functional dichotomy of ILC3 subsets (NCR+ versus NCR−) is governed by metabolic plasticity driven by the mTOR-glycolysis axis, which dictates the phenotypic transition from protective IL-22 production to a pathogenic IFN-γ-secreting profile during intestinal inflammation (84). During the stable phase of the disease, NCR+ ILC3s utilize moderate glycolysis to support RORγt expression and IL-22 secretion, thereby facilitating tissue repair. However, under the metabolic constraints of chronic inflammation—such as hypoxia or glucose deprivation—heightened glycolytic activity may trigger the conversion of ILC3s into a pathogenic ‘ex-ILC3’ phenotype. These cells switch to IFN-γ production, effectively shifting from protective mediators to inflammatory amplifiers. Conversely, during the acute phase of UC, NCR− ILC3s recruit massive neutrophil infiltration and induce pro-inflammatory chemokine release via IL-17, further exacerbating mucosal tissue injury (85).

### 3.4 CD4+T cells

T cell populations serve as the principal orchestrators of the intestinal adaptive immune landscape, conferring a requisite spatiotemporal precision to mucosal immunosurveillance (86). This role is fundamental to sustaining the delicate equilibrium between systemic immune tolerance and protective, defensive inflammation (87, 88). The intestinal T-cell compartment is characterized by a sophisticated hierarchical architecture, with a phenotypic spectrum spanning from innate-like intraepithelial lymphocytes (IELs) providing rapid mucosal cytotoxicity to specialized effector cell populations residing in the lamina propria (LPLs) (89) (90). Within the pathological microenvironment of UC, the mTORC1 and mTORC2 signaling pathways serve as central molecular switches that cooperatively orchestrate CD4+ T cell differentiation and plasticity. The metabolic remodeling mediated by these pathways is a fundamental determinant of intestinal immune homeostasis. Specifically, mTORC1 acts in concert with HIF-1α to augment glycolytic flux, thereby driving the pro-inflammatory polarization of Th1 and Th17 cells (91). Conversely, mTORC2 specifically induces Th2 cell development via the Akt signaling axis while finely tuning the suppressive capacity and stability of regulatory T (Treg) cells. The resulting immunometabolic dysregulation, triggered by aberrant mTOR signaling, is characterized by the pathogenic expansion of effector T cells—driven by an over-reliance on glycolysis—and a pathological shift of the Th17/Treg balance toward a pro-inflammatory phenotype under severe metabolic stress. These alterations underpin the persistent mucosal immune activation and non-resolving inflammation observed in UC patients (92, 93).

The divergent differentiation of these CD4+ T cells in the intestinal microenvironment of UC—most notably the pivotal Th17/Treg immunological axis—is synergistically orchestrated by the commensal metabolome and site-specific transcriptional networks, thereby sustaining the delicate equilibrium of the intestinal immune microenvironment (94, 95). Research indicates that the mTOR-HIF-1α signaling axis dictates the reciprocal interconversion between Th17 and Treg cells through the precise modulation of glycolytic flux (96). While mTORC1-mediated induction of aerobic glycolysis is indispensable for the anabolic demands and epigenetic remodeling associated with Th17 pathogenicity, its hyperactivation paradoxically destabilizes the Foxp3+ suppressive program (97, 98). This metabolic bifurcation is dictated by the strategic orchestration of glycolytic enzymes and nutrient transporters, thereby fueling Th17 lineage commitment at the expense of Treg-mediated immune tolerance (99). In the pathogenesis of UC, Wang et al. identified the mTOR-HIF1α axis as a critical metabolic fulcrum that fuels the intestinal inflammatory cascade. This axis exacerbates disease by skewing CD4+ T cell fate toward glycolysis-dependent Th17 pathogenicity while simultaneously undermining the suppressive integrity of the Treg pool—a pathological process that can be reversed through mTOR inhibition (100). Liu et al. demonstrated that Astragalus polysaccharides (APS) attenuate mTOR/HIF-1α-mediated glycolytic flux, thereby steering T-cell differentiation toward a Foxp3-driven immunotolerant phenotype and ultimately ameliorating colonic inflammation (101). Yang et al. proposed that GPR120 signaling effectively redirects CD4+ T cell fate from pathogenic Th17 infiltration toward an IL-10-producing regulatory phenotype by suppressing the mTOR-driven glycolytic program, thereby restoring intestinal homeostasis (102). In summary, the mTOR-glycolysis axis serves as a metabolic conduit that skews the immune landscape toward a pro-inflammatory state. By empowering pathogenic Th17 lineage at the cost of Treg stability, this axis sustains a metabolic bias that prevents the healing of intestinal mucosal lesions (103).

In conclusion, On the pro-inflammatory axis, mTORC1 activation orchestrates the metabolic reprogramming of Th17 cells, M1-polarized macrophages, and neutrophils. Notably, neutrophils harness a glycolytic burst to fuel their key effector functions, including chemotaxis, degranulation, and the release of NETs. This process is mediated by the mTOR/HIF-1α signaling cascade, with the magnitude of glycolytic induction positively correlating with the severity of acute tissue injury. Conversely, on the homeostatic and reparative front, mTORC2 signaling is indispensable for maintaining the metabolic flexibility and immunosuppressive potency of Treg cells and M2 macrophages, thereby indirectly fortifying intestinal barrier integrity and facilitating mucosal healing. although the mTOR-glycolysis axis represents a foundational pillar for advancing immunometabolic therapeutic strategies in UC (**Table 2**), the inherent spatiotemporal complexity and cellular heterogeneity of the intestinal landscape remain critical determinants. Given the immense clinical potential yet to be tapped, this axis should be strategically integrated into the development of precisely targeted interventions.

**Table 2:** The role and mechanism of mTOR and glycolysis in regulating immune cells in the intestinal microenvironment of UC.

Research approach	Animal model	Target cell	Research mechanism	References
*in vivo*	DSS-induced Colitis	Macrophages, Neutrophils	Glucocorticoids drive intestinal inflammation by activating epithelial-specific mTORC1, which in turn modulates macrophage and neutrophil responses.	(62)
*in vivo*	DSS-induced Colitis	Macrophages, Neutrophils	Biomimetic Pd@M nanozymes modulate macrophage polarization and neutrophil infiltration by neutralizing oxidative stress and thwarting glycolytic reprogramming.	(63)
*in vivo* and *in vitro*	DSS-induced colitis	Macrophages	Dioscin modulates the mTORC1/HIF-1α and mTORC2/PPAR-γ signaling axes to suppress aerobic glycolysis, thereby facilitating M2 macrophage polarization.	(72)
*in vivo* and *in vitro*	DSS-induced Colitis	Macrophages	Tiliroside reprograms macrophage polarization by targeting HIF-1α-dependent aerobic glycolysis.	(74)
*in vivo* and *in vitro*	DSS-induced Colitis	Macrophages	XHCF orchestrates the M1-to-M2 macrophage phenotypic switch through AMPK-mediated metabolic reprogramming, which is characterized by the suppression of HK2-dependent glycolysis.	(75)
*in vitro*	–	Th17 cells, Treg cells	The mTOR/HIF-1α signaling axis drives the pathogenesis of ulcerative colitis (UC) by promoting glycolysis-dependent Th17 polarization and impairing Treg fitness; notably, this pathological process can be reversed through mTOR inhibition.	(100)
*in vivo*	DSS-induced Colitis	Treg cells	Astragalus polysaccharide (APS) modulates Treg cell responses by suppressing aerobic glycolysis and the mTOR/HIF-1α signaling axis.	(101)
*in vivo*	DSS-induced Colitis、Salmonella typhimurium-induced infectious colitis、Rag(-/-) mice	CD4+ T cells	GP420 orchestrates an mTOR-dependent metabolic-transcriptional axis that synergistically elevates Blimp1 expression and reinforces glycolytic flux, thereby augmenting the IL-10-producing capacity of CD4+ T cells.	(102)
*in vivo* and *in vitro*	DSS-induced Colitis	Macrophages	The Myo1F/Integrin-αVβ3/mTOR axis drives macrophage-mediated colonic inflammation by coupling intercellular adhesion to pro-inflammatory M1 polarization.	(104)
*in vivo*	DSS-induced Colitis	Macrophages	Apremilast perturbs mucosal macrophage immunity by modulating the PDE4-dependent crosstalk of PKA/Epac with the NF-κB, PI3K-mTOR, and JAK-STAT signaling cascades.	(105)
*in vivo* and *in vitro*	DSS-induced Colitis	Macrophages	Cinnamaldehyde attenuates UC by suppressing ROS generation, AKT/mTOR signaling, and NLRP3 inflammasome activation, while simultaneously downregulating the expression of miR-21 and miR-155 within the colon and macrophages.	(106)
*in vitro*	–	Macrophages	Macrophage MMP12 expression is governed by a glycolysis-dependent metabolic crosstalk between the AMPK and mTORC1 pathways.	(107)
*in vivo* and *in vitro*	DSS-induced Colitis	Dendritic cells, Th17 cells	The mTORC2 signaling axis drives pro-inflammatory Th17 responses in UC by orchestrating Rac1/Cdc42-mediated cytoskeletal remodeling and the subsequent migration of dendritic cells.	(108)
*in vivo* and *in vitro*	DSS-induced Colitis	Macrophages	ES100-PIP/GA NC reprograms the macrophage polarization landscape by augmenting mTOR-mediated M2 polarization while simultaneously attenuating HIF-1α-dependent M1 activation.	(109)
*in vivo* and *in vitro*	DSS-induced Colitis	Macrophages, Intestinal epithelial cells	D-mannose modulates macrophage M1/M2 polarization by activating the AMPK/mTOR signaling axis while directly upregulating epithelial tight junction proteins to reinforce barrier integrity.	(110)
*in vivo*	DSS-induced Colitis	Macrophages	Bangle (Zingiber purpureum Rosc.) Extractameliorates colonic inflammation by modulating the AMPK/mTOR/NFκB signaling axis, thereby reducing macrophage infiltration in the inflamed colon.	(111)
*in vivo*	SAMHD1^mKO^ mice	Macrophages	SAMHD1 deficiency unleashes the mTOR signaling pathway, where aberrant mTOR hyperactivation suppresses the MITF-CTSD-mediated lysosomal homeostatic program in macrophages.	(112)
*in vivo*	DSS-induced Colitis	Macrophages	Berberine promotes M2 macrophage polarization by suppressing the mTOR/HIF-1α axis in a PPAR-γ-dependent manner, thereby augmenting oxidative phosphorylation.	(113)
*in vivo*	DSS-induced Colitis	Macrophages	Wenyang Decoction orchestrates the macrophage phenotypic switch from M1 to M2 and mitigates oxidative stress by suppressing the PI3K/AKT/mTOR/HIF-1α signaling axis.	(114)
*in vivo* and *in vitro*	DSS-induced Colitis	Macrophages	Dehydrocorydaline (DHC) blunts glycolytic flux by dual-targeting the enzymatic activity and formylation of GAPDH, thereby suppressing lactate production and subsequent macrophage activation.	(115)
*in vivo* and *in vitro*	DSS-induced Colitis	Macrophages	Shikonin abrogates aerobic glycolysis by suppressing the PKM2-HIF-1α metabolic axis, thereby attenuating macrophage-mediated inflammatory responses.	(116)
*in vivo*	DSS-induced Colitis	Macrophages	ADMSCs orchestrate a bidirectional Succinate-PGE2 feedback loop to dismantle the HIF-1α-dependent glycolytic engine in macrophages, thereby reinstating immune homeostasis.	(117)
*in vivo* and *in vitro*	DSS-induced Colitis	Macrophages	HMGB1 triggers a metabolic switch from fatty acid oxidation to glycolysis through the transcriptional repression of Cpt1a, thereby driving pro-inflammatory M1 macrophage polarization.	(118)
*in vivo* and *in vitro*	DSS-induced Colitis	Macrophages	Ginsenoside Rg1-primed ADSCs attenuate pro-inflammatory glycolytic flux in macrophages via the exosomal miR-574-3p/RAS axis.	(119)
*in vivo*	DSS-induced Colitis	Macrophages	Total flavonoids from Cynanchum chinense synergistically orchestrate Keap1-Nrf2-mediated mucosal barrier repair, suppress glycolysis-driven macrophage metabolic reprogramming, and reshape gut microecological homeostasis.	(120)
*in vivo*	DSS-induced Colitis、Fats(-/-) mice	Macrophages	FATS attenuates pro-inflammatory macrophage polarization and mucosal injury by modulating HIF-1α-mediated glycolytic flux.	(121)
*in vivo* and *in vitro*	DSS-induced Colitis	Macrophages	IPA orchestrates a macrophage M1-to-M2 phenotypic switch in colitis by suppressing JNK/MAPK-mediated glycolysis and augmenting PPAR-γ-dependent lipid metabolism.	(122)
*in vivo*、*in vitro* and clinical samples	DSS-induced Colitis	Macrophages	EBV/MHV-68 infection hijacks macrophage glucose metabolism to elicit NLRP3/GSDMD-mediated pyroptosis.	(123)
*in vivo* and *in vitro*	DSS-induced Colitis	Macrophages	Impaired PDH-dependent glucose oxidation fuels M1 macrophage-mediated colitis under thiamine deficiency.	(124)
*in vivo*	DSS-induced Colitis	Macrophages	DOPS activates the SENP1-SIRT3 axis to curtail macrophage glycolysis and foster M2 polarization.	(125)
*in vivo*	DSS-induced Colitis	Neutrophils	Glutamine suppresses the PI3K/Akt/mTOR signaling axis, thereby abrogating neutrophil infiltration and mitigating associated colonic oxidative damage.	(126)
*in vivo* and clinical samples	DSS-induced Colitis	Neutrophils	Spleen tyrosine kinase (Syk) orchestrates neutrophil effector functions—including NETosis and oxidative stress—via the mTOR/Rubicon-dependent autophagy pathway.	(127)
*in vivo*	DSS-induced Colitis	Neutrophils	Atractylenolide-I suppresses neutrophil-derived S100A9 release and subsequently activates the AMPK/mTOR signaling axis to reinstate tight junction protein expression.	(128)
*in vivo*	DSS-induced Colitis	Th17 cells, Treg cells	Resveratrol dampens the HIF-1α/mTOR/STAT3 signaling cascade to orchestrate Th17/Treg homeostasis.	(129)
*in vivo*	DSS-induced Colitis	Th17 cells, Treg cells	BL-hIL-10 rectifies the Th17/Treg imbalance by targeting a convergent mTOR-STAT3-HIF-1α signaling network.	(130)
clinical sample	–	Th17 cells, Treg cells	CCR6 signaling orchestrates iTreg-to-Th17 lineage plasticity via the Akt/mTOR/STAT3 axis.	(131)
*in vivo* and clinical sample	DSS-induced Colitis	Th17 cells	Hyperactivated epithelial mTORC1 drives the recruitment of pathogenic Th17 cells by initiating a STAT3-dependent COX-2 transcriptional program.	(132)
*in vivo*	DSS-induced Colitis	Th17 cells	The synergistic combination of empagliflozin and metformin activates the AMPK signaling pathway, thereby suppressing the mTOR/NLRP3 inflammasome axis and subsequent Th17 cell polarization.	(133)
*in vivo*	DSS-induced Colitis	Th17 cells, Treg cells	PD-L1-EVs rectify Th17/Treg dyshomeostasis and multidimensional mucosal injury by targeting the PI3K/Akt/mTOR signaling axis.	(134)
*in vivo*	DSS-induced Colitis	Th17 cells, Treg cells	Cinnamtannin D1 re-establishes Th17/Treg homeostasis by engaging the AMPK/mTOR signaling axis.	(135)
*in vivo*	DSS-induced Colitis	Th17 cells, Treg cells	Butyrate recalibrates Th17/Treg immune homeostasis through cAMP-PKA/mTOR-mediated immunomodulation.	(136)
*in vivo* and *in vitro*	DSS-induced Colitis	Th17 cells, Treg cells, Intestinal epithelial cells	Artesunate promotes HMGCS2-dependent ketogenesis by activating the AMPK/mTOR signaling axis, thereby restoring Th17/Treg homeostasis and intestinal epithelial barrier integrity.	(137)
*in vivo* and *in vitro*	DSS-induced Colitis	Th17 cells, Treg cells	Senkyunolide selectively inhibits Th17 cell glycolysis and restores Th17/Treg homeostasis by triggering the PHD2-mediated proteasomal degradation of HIF-1α.	(138)
*in vivo* and *in vitro*	DSS-induced Colitis	Th17 cells, Treg cells	DIM suppresses Th17 pathogenicity and bolsters Foxp3 stability via the lactate-STAT3 and HIF-1α/TIP60 signaling cascades.	(139)
*in vivo*	DSS-induced Colitis、TNBS-induced Colitis	Th17 cells, Treg cells	D5, a novel cinnamyl alcohol ester derivative, selectively suppresses Th17-driven glycolysis and reinstates the Th17/Treg immune balance through the covalent activation of PKM2 at the Cys424 residue.	(140)
*in vivo* and *in vitro*	DSS-induced Colitis	Th17 cells, Treg cells	Inhibition of HSP90β triggers GLUT1 ubiquitination and suppresses glycolysis, which subsequently drives Foxp3 epigenetic remodeling via the lactate-STAT5-TET2 axis, ultimately inducing Th17-to-Treg transdifferentiation.	(141)
*in vivo* and *in vitro*	DSS-induced Colitis	Th17 cells, intestinal epithelial cells	Phascolarcto bacterium depletes luminal succinate, thereby attenuating SUCNR1-mediated glycolytic remodeling in both epithelial and Th17 cells.	(142)
*in vivo*	DSS-induced Colitis	Dendritic cells, Intestinal epithelial cells, CD4+ T cells	Colon-targeted delivery of MFGE8-enriched ERC-derived exosomes reconfigures intestinal epithelial cell survival and CD4+ T cell proliferation while suppressing dendritic cell maturation through the PI3K/AKT/mTOR signaling axis.	(143)

## 4 Metabolic rewiring of the mTOR-glycolysis axis in intestinal epithelial cells: from barrier breakdown to impaired mucosal repair

The mTOR signal pathway functions as a central metabolic rheostat within the intestinal epithelium, exquisitely orchestrating the coupling of luminal nutrient sensing with mucosal regeneration kinetics and autophagy-driven proteostasis to safeguard intestinal barrier integrity (137, 144). The metabolic landscape of intestinal epithelial cells (IECs) is governed by a finely tuned glycolytic program that functions as a fundamental bioenergetic checkpoint, orchestrating mucosal niche remodeling and adaptive barrier responses under both homeostasis and inflammatory stress (145). Serving as an integrated ‘‘metabolic-effector’’ hub in IECs, the mTOR-glycolytic signaling axis couples nutrient sensing with pathogenic metabolic reprogramming upon aberrant activation, ultimately dictating the phenotypic transition from mucosal homeostasis to inflammatory barrier collapse (146). Inflammation is characterized by a metabolic switch that favors aerobic glycolysis and lactate production. Specifically, apical HK2 expression serves as a hallmark of severe metabolic remodeling in epithelial cells, heralding imminent mucosal ulceration or erosion. This suggests that dysregulated epithelial HK2 expression acts as a driver of exacerbated intestinal inflammation (28). Concurrently, PDK2 is significantly upregulated in patients with active UC, where its overexpression signals profound mitochondrial dysfunction within IECs (147). Crucially, these pathological processes are triggered by the activation of the upstream mTOR signaling pathway. The resulting cellular metabolic derangement within the microenvironment ultimately culminates in the collapse of the mucosal barrier.

It’s worth noting that various agents, including halogenated acylaminopyridine-based molecules (P2281) (148), the flavonoid derivative 3”,4”,5”-trihydroxyflavone (THF; NJK16003) (149), heat shock factor 2 (HSF2) (150), Metrnl (151), and inulin-type fructans (CP-A) (152), function as potent mTOR inhibitors. These candidates safeguard the mucosal barrier by antagonizing the mTOR signal pathway, which subsequently attenuates IEC apoptosis and potentiates autophagy to facilitate tissue repair. Concurrently, Palmatine (PAL) (153) and barley leaf (154) have been shown to restore the IEC barrier by suppressing glycolytic metabolic reprogramming, thereby ameliorating the clinical symptoms of UC. Despite extensive characterization of the mTOR signal pathway and glucose metabolism as standalone regulators of IEC homeostasis (**Table 3**), evidence regarding their functional crosstalk within a unified mTOR-glycolysis axis remains conspicuously scarce.

**Table 3:** The role and mechanism of mTOR and glycolysis in regulating intestinal epithelial cells in the intestinal microenvironment of UC.

Research approach	Animal model	Target cell	Research mechanism	References
*in vivo* and *in vitro*	DSS-induced Colitis	Macrophages, Intestinal epithelial cells	D-mannose activates the AMPK/mTOR signaling axis to modulate macrophage M1/M2 polarization while simultaneously upregulating tight junction proteins in epithelial cells directly.	(110)
*in vivo* and *in vitro*	DSS-induced Colitis	Th17 cells, Treg cells, Intestinal epithelial cells	Artesunate promotes HMGCS2-dependent ketogenesis by activating the AMPK/mTOR signaling axis, thereby restoring Th17/Treg homeostasis and intestinal epithelial barrier integrity.	(137)
*in vivo* and *in vitro*	DSS-induced Colitis	Th17 cells, Intestinal epithelial cells	Phascolarcto bacterium depletes luminal succinate, thereby attenuating SUCNR1-mediated glycolytic remodeling in both epithelial and Th17 cells.	(142)
*in vivo*	DSS-induced Colitis	Dendritic cells, Intestinal epithelial cells, CD4+ T cells	Colon-targeted delivery of MFGE8-enriched ERC-derived exosomes reconfigures intestinal epithelial cell survival and CD4+ T cell proliferation while suppressing dendritic cell maturation through the PI3K/AKT/mTOR signaling axis.	(143)
*in vivo* and *in vitro*	DSS-induced Colitis	Intestinal epithelial cells	P2281, a novel mTOR inhibitor, attenuates mTOR-driven T-cell activation and safeguards the intestinal epithelial crypt architecture against inflammatory destruction.	(148)
*in vivo* and *in vitro*	DSS-induced Colitis	Intestinal epithelial cells	NJK16003 safeguards intestinal epithelial cells against apoptosis and inflammatory damage by harnessing an mTOR-dependent autophagy program.	(149)
*in vivo*、*in vitro* and clinical samples	DSS-induced Colitis、HSF2(-/-) mice	Intestinal epithelial cells	Butyrate-mediated epigenetic induction of HSF2 promotes intestinal epithelial autophagy via the PI3K/Akt/mTOR signaling axis.	(150)
*in vivo*	DSS-induced Colitis、Metrnl(-/-) mice	Intestinal epithelial cells	Metrnl-dependent activation of the AMPK/mTOR/p70S6K signaling axis sustains protective autophagy in intestinal epithelial cells.	(151)
*in vivo* and *in vitro*	TNBS-induced Colitis	Intestinal epithelial cells	CP-A suppresses the mTOR/p70S6K signaling axis to attenuate the production of pro-inflammatory cytokines in intestinal epithelial cells.	(152)
*in vivo* and *in vitro*	DSS-induced Colitis	Intestinal epithelial cells	Palmatine reinforces intestinal barrier integrity by suppressing excessive glycolysis in intestinal epithelial cells through the direct targeting and upregulation of ENO3.	(153)
*in vivo*	DSS-induced Colitis	Intestinal epithelial cells	Barley leaf drives inosine enrichment, which activates the A(2A)R/PPARγ signaling axis and orchestrates cellular metabolic reprogramming to reinforce the intestinal epithelial barrier.	(154)
*in vivo*	DSS-induced Colitis、PBLD(IEC-/-) mice	Intestinal epithelial cells	Rapamycin fortifies the intestinal epithelial barrier in ulcerative colitis by suppressing the mTOR/TFEB/PBLD signaling axis.	(155)
*in vivo*	DSS-induced Colitis	Intestinal epithelial cells	During mucosal healing, IECs undergo a rapid metabolic switch toward robust glycolytic ATP production, driven by mitochondrial fusion and augmented respiratory flux, to bridge the bioenergetic deficit elicited by colitis.	(156)
*in vivo*、*in vitro* and clinical samples	(Crt-/-) mice	Intestinal epithelial cells	Deficiency of the creatine transporter CRT disrupts intestinal epithelial energy homeostasis and triggers glycolytic stress.	(157)
*in vivo*、*in vitro* and clinical samples	DSS-induced Colitis、Piezo1^ΔIEC^ mice	Intestinal epithelial cells	Upregulated epithelial Piezo1 triggers ferroptosis-dependent barrier dysfunction by modulating the AMPK/mTOR signaling axis.	(158)
*in vivo*	DSS-induced Colitis	Intestinal epithelial cells	Pingwei San attenuates the PI3K/AKT/mTOR signaling cascade, thereby augmenting autophagy-mediated mucosal protection in intestinal epithelial cells.	(159)
*in vivo*、*in vitro* and clinical samples	DSS-induced Colitis、Klf5^ΔIEC^ rats	Intestinal epithelial cells	GPR35 orchestrates a KLF5-centered transcriptional program via the PI3K-AKT-mTOR axis to govern the proliferation and migration of intestinal epithelial cells.	(160)

## 5 Integrated cross-cell-type analysis

Beyond its role as a mere structural conduit, the intestinal epithelium operates in concert with mucosal immune cell populations as an interdependent functional entity. Within this nexus, reciprocal molecular signaling and metabolic feedback loops exquisitely orchestrate the spatiotemporal transition between homeostatic immunosurveillance and effector inflammatory responses (161, 162).

### 5.1 Intestinal epithelial cells and macrophages

The homeostatic tethering between intestinal epithelial cells and resident lamina propria macrophages characterizes a distinct, niche-dependent crosstalk (163, 164). Specifically, epithelium-derived instructive signals, such as CSF1 and TGF-β, reprogram macrophages toward a non-inflammatory, ‘pro-resolving’ phenotype; in turn, these macrophages provide paracrine trophic support to reciprocally modulate epithelial progenitor proliferation and mucosal wound healing (165). Emerging evidence indicates that the mTORC1 signaling axis orchestrates a functional bifurcation of the mucosal inflammatory secretome, driving intestinal epithelial Caco-2 cells and THP-1-derived macrophages to exhibit distinct, if not diametrically opposed, cytokine expression patterns under identical metabolic stimuli (166). At the metabolic level, alcohol-induced mitochondrial impairment in intestinal epithelial cells precipitates a dose-dependent bioenergetic crisis, necessitating a compensatory glycolytic shift that culminates in barrier collapse. This structural failure, in synergy with the subsequent translocation of microbial PAMPs and epithelium-derived metabolic stress signals, collectively orchestrates the pro-inflammatory reprogramming of intestinal macrophages (167). Taken together, these findings underscore that the mTORC1-glycolysis signaling axis not only coordinates the symbiotic tethering between intestinal epithelial cells and pro-resolving macrophages under homeostasis but also potentially dictates the phenotypic transition toward a pathological pro-inflammatory landscape by driving a ‘metabolic-secretory’ bifurcation in response to mitochondrial stress and glycolytic reprogramming.

### 5.2 Intestinal epithelial cells and T cells

A bidirectional crosstalk exists between intestinal epithelial cells (IECs) and mucosal CD4+ T cell subsets. Evidence demonstrates that IECs, acting as unconventional MHC II-expressing antigen-presenting cells, fine-tune bacteria-reactive CD4+ T cell responses by differentially modulating effector and regulatory Th subsets. Conversely, CD4+ T cell-derived cytokines—most notably IL-22 produced by Th17/Th22 cells—activate STAT3 signaling to reinforce epithelial barrier integrity through the upregulation of antimicrobial peptides and tight junction component (168, 169). Focusing on the mTOR signaling pathway, Yingshu Luo et al. demonstrated that the SARS-CoV-2 spike protein exploits the CEACAM5-Galectin-9 interface to dismantle the homeostatic crosstalk between the intestinal epithelium and T cells. In this context, aberrantly released Galectin-9 acts as a ‘molecular wedge’ that impairs mTOR-mediated metabolic fitness in resident T cells, ultimately precipitating a feed-forward loop of mucosal barrier disintegration (170). Regarding metabolic rewiring, Daniela Parada Venegas highlights that SCFAs orchestrate a ‘‘metabolic-epigenetic’’ rheostat, channeling epithelial bioenergetics toward oxidative phosphorylation to foster a glucose-sparing microenvironment. This niche selectively facilitates the HDAC inhibition-dependent expansion of regulatory T cells (Tregs) while curtailing the metabolic surge of pathogenic T cells, ultimately reinstating mucosal harmony in the context of IBD (171). Collectively, these insights suggest that the reciprocal crosstalk between IECs and CD4+ T cells is evidently orchestrated by an mTOR-glycolytic metabolic rheostat. This axis appears to function as a fundamental nexus that integrates immunological signals and bioenergetic cues to sustain mucosal homeostasis.

### 5.3 Intestinal epithelial cells and other immune cells

Far from being mere static physical barriers, IECs function as critical ‘immune sentinels.’ Upon mucosal perturbation, IECs mount a localized, bioactive secretome comprising chemokines and cytokines, thereby precisely orchestrating the rapid spatiotemporal influx of neutrophils (172, 173). Acting as a pivotal immunomodulatory interface, the intestinal epithelium transduces luminal microbial cues into potent paracrine signals, thereby orchestrating B cell class switch recombination and IgA plasmablast differentiation. This regulatory cascade ensures the targeted deployment of secretory IgA (sIgA), a prerequisite for sustaining commensal homeostasis (174, 175). Furthermore, the mucus-mucin matrix synthesized by goblet cells and IECs functions as a dynamic immunomodulatory scaffold. It actively modulates the mucosal niche by facilitating DCs sampling via specialized goblet cell-associated antigen passages (GAPs), while simultaneously conferring a tolerogenic imprint on these sentinel cells through MUC2-mediated signaling pathways (176). While the reciprocal signaling between the intestinal epithelium and diverse immune subsets has been preliminarily delineated, the mechanistic contribution of the mTOR-dependent glycolytic pathway to this homeostatic interplay remains elusive, representing a critical knowledge gap within the contemporary immunometabolic paradigm.

## 6 Conclusion and prospect

The burgeoning field of immunometabolism has emerged as a pivotal lens for reappraising the multifactorial etiology of UC, centered on the intricate interplay between bioenergetic pathways and immune dysregulation (177). Compelling evidence suggests that the bioenergetic synergy between mTOR pathway activation and glycolytic flux has emerged as a defining hallmark of UC. This interplay serves as a critical molecular determinant, governing both the inflammatory trajectory of the colonic mucosa and its responsiveness to therapeutic intervention (178, 179). At the epicenter of the immunometabolic network, mTOR functions as the master orchestrator that synchronizes intestinal immune homeostasis with bioenergetic balance (180, 181). In this regulatory hierarchy, the glycolytic pathway serves as a pivotal downstream effector, fine-tuning the functional responsiveness of both IECs and immune cell subsets through metabolic reprogramming (182, 183). While clinically approved mTOR inhibitors have been widely utilized in the management of renal transplantation (184), thalassemia (185), and diverse malignancies (186, 187) (**Table 4**), their application in UC remains clinically restricted. To date, major global regulatory authorities—including the FDA, EMA, and NMPA—have yet to formally approve any mTOR inhibitor as a standalone therapeutic modality for the treatment of UC. However, a comprehensive synthesis that integrates the regulatory influence of the mTOR-glycolysis axis within the intestinal immune microenvironment remains elusive, particularly regarding the intricate metabolic crosstalk between immune cells and IECs. By situating this signaling axis at the immunometabolic interface, we delineate the bioenergetic heterogeneity and interaction dynamics among diverse cellular populations. These insights offer a transformative paradigm in the pathobiology of ulcerative colitis, underscoring the immense potential of targeting metabolic checkpoints as a novel avenue for future therapeutic intervention.

**Table 4:** Clinically approved mTOR inhibitors.

Drug name	Approval number	Adaptation to symptoms	References
Sirolimus	NDA 021083 NDA 021110	Organ rejection after kidney transplantation; lymphangioleiomyomatosis	(188, 189)
Temsirolimus	NDA 022088	Advanced renal cell carcinoma	(190)
Everolimus	NDA 022334 NDA 203985 NDA 021560	Tuberous sclerosis syndrome; locally advanced or metastatic pancreatic neuroendocrine tumors that cannot be resected; liver transplantation	(191–193)
nab-Sirolimus	NDA 213174	Locally advanced, unresectable or metastatic malignant perivascular epithelioid cell tumor	(194)

However, prominent side effects, including oral mucositis and myelosuppression, combined with the drawbacks of non-selective systemic immunosuppression, largely preclude the widespread clinical application of conventional mTOR inhibitors for UC treatment. Functioning as the central immunometabolic rheostat within the UC microenvironment, mTOR integrates intraluminal nutrient availability with systemic inflammatory cues. This integration allows it to precisely orchestrate the functional polarization of mucosal immune subsets and the homeostatic renewal of the intestinal epithelium, thereby serving as a master arbiter of intestinal barrier integrity and immunological tolerance (195). The mTOR-glycolysis axis deciphers intraluminal cues to fulfill the bioenergetic requirements of mucosal immune cells. Aberrant overactivation of this circuitry precipitates a homeostatic shift from immune tolerance toward inflammatory exacerbation, particularly during the pathogenesis of UC. Notably, pathological hyperactivation of mTOR-driven glycolytic programs selectively provides the metabolic momentum for the expansion of pro-inflammatory effectors, such as Th17 and M1 cells, while simultaneously antagonizing the lineage stability and immunosuppressive potency of Tregs within the inflamed mucosal niche (53). Future strategies aiming to harness the mTOR pathway for microenvironmental restoration in UC must transition from broad systemic immunosuppression to targeted local reprogramming. Firstly, innovative drug delivery modalities, including rectal administration and pH-dependent colonic release, should be employed to restrict pharmacological activity to the intestinal lamina propria. This spatial targeting is crucial to avoid systemic side effects and prevent the impairment of mucosal healing. Secondly, the development of selective mTOR pathway modulators, particularly mTORC1-specific inhibitors, represents a promising avenue to achieve therapeutic efficacy while preserving the vital regenerative potential of the intestinal epithelium. In addition, the inherent multidimensional complexity and cellular heterogeneity of the intestinal environment pose formidable obstacles to deconstructing the cell-type-specific nuances of the mTOR-glycolysis axis during UC progression. To bridge these knowledge gaps, future endeavors must harness interdisciplinary synergies—integrating single-cell and spatial multi-omics—to chart a comprehensive blueprint of how this immunometabolic axis dictates intestinal homeostasis and drives disease evolution. Such efforts will be pivotal in elucidating spatiotemporal immunometabolic crosstalk and identifying stage-specific therapeutic vulnerabilities within the UC microenvironment.

## References

[1] Ungaro R Mehandru S Allen PB Peyrin-Biroulet L Colombel JF . Ulcerative colitis. Lancet. (2017) 389:1756–70. doi: 10.1016/S0140-6736(16)32126-2 27914657 PMC6487890

[2] Ng SC Shi HY Hamidi N Underwood FE Tang W Benchimol EI . Worldwide incidence and prevalence of inflammatory bowel disease in the 21st century: a systematic review of population-based studies. Lancet. (2017) 390:2769–78. doi: 10.1016/S0140-6736(17)32448-0 29050646

[3] Kobayashi T Siegmund B Le Berre C Wei SC Ferrante M Shen B . Ulcerative colitis. Nat Rev Dis Primers. (2020) 6:74. doi: 10.1038/s41572-020-0205-x 32913180

[4] Le Berre C Honap S Peyrin-Biroulet L . Ulcerative colitis. Lancet. (2023) 402:571–84. doi: 10.1016/S0140-6736(23)00966-2 37573077

[5] Saxton RA Sabatini DM . mTOR signaling in growth, metabolism, and disease. Cell. (2017) 168:960–76. doi: 10.1016/j.cell.2017.03.035 28283069 PMC5394987

[6] Powell JD Pollizzi KN Heikamp EB Horton MR . Regulation of immune responses by mTOR. Annu Rev Immunol. (2012) 30:39–68. doi: 10.1146/annurev-immunol-020711-075024 22136167 PMC3616892

[7] Bonetti L Horkova V Grusdat M Longworth J Guerra L Kurniawan H . A Th17 cell-intrinsic glutathione/mitochondrial-IL-22 axis protects against intestinal inflammation. Cell Metab. (2024) 36:1726–44.e10. doi: 10.1016/j.cmet.2024.06.010 38986617

[8] Guo H Gao J Qian Y Wang H Liu J Peng Q . miR-125b-5p inhibits cell proliferation by targeting ASCT2 and regulating the PI3K/AKT/mTOR pathway in an LPS-induced intestinal mucosa cell injury model. Exp Ther Med. (2021) 22:838. doi: 10.3892/etm.2021.10270 34149884 PMC8210225

[9] Lunt SY Vander Heiden MG . Aerobic glycolysis: meeting the metabolic requirements of cell proliferation. Annu Rev Cell Dev Biol. (2011) 27:441–64. doi: 10.1146/annurev-cellbio-092910-154237 21985671

[10] Soto-Heredero G Gómez de Las Heras MM Gabandé-Rodríguez E Oller J Mittelbrunn M . Glycolysis - a key player in the inflammatory response. FEBS J. (2020) 287:3350–69. doi: 10.1111/febs.15327 32255251 PMC7496292

[11] He L Cho S Blenis J . mTORC1, the maestro of cell metabolism and growth. Genes Dev. (2025) 39:109–31. doi: 10.1101/gad.352084.124 39572234 PMC11789495

[12] Ragupathi A Kim C Jacinto E . The mTORC2 signaling network: targets and cross-talks. Biochem J. (2024) 481:45–91. doi: 10.1042/BCJ20220325 38270460 PMC10903481

[13] Jiang C Tan X Liu N Yan P Hou T Wei W . Nutrient sensing of mTORC1 signaling in cancer and aging. Semin Cancer Biol. (2024) 106-107:1–12. doi: 10.1016/j.semcancer.2024.08.001 39153724

[14] Khan W Zeb A Malik MFA Wahid M Mandal RK Babegi AS . FGF21 affects the glycolysis process via mTOR-HIF1α axis in hepatocellular carcinoma. Cell Signal. (2025) 126:111522. doi: 10.1016/j.cellsig.2024.111522 39580062

[15] Toschi A Lee E Gadir N Ohh M Foster DA . Differential dependence of hypoxia-inducible factors 1 alpha and 2 alpha on mTORC1 and mTORC2. J Biol Chem. (2008) 283:34495–9. doi: 10.1074/jbc.C800170200 18945681 PMC2596400

[16] Lee S Byun JK Kim NY Jin J Woo H Choi YK . Melatonin inhibits glycolysis in hepatocellular carcinoma cells by downregulating mitochondrial respiration and mTORC1 activity. BMB Rep. (2022) 55:459–64. doi: 10.5483/BMBRep.2022.55.9.177 35651333 PMC9537022

[17] Düvel K Yecies JL Menon S Raman P Lipovsky AI Souza AL . Activation of a metabolic gene regulatory network downstream of mTOR complex 1. Mol Cell. (2010) 39:171–83. doi: 10.1016/j.molcel.2010.06.022 20670887 PMC2946786

[18] Zhao X Jiang P Deng X Li Z Tian F Guo F . Inhibition of mTORC1 signaling sensitizes hepatocellular carcinoma cells to glycolytic stress. Am J Cancer Res. (2016) 6:2289–98 27822418 PMC5088292

[19] Jhanwar-Uniyal M Amin AG Cooper JB Das K Schmidt MH Murali R . Discrete signaling mechanisms of mTORC1 and mTORC2: Connected yet apart in cellular and molecular aspects. Adv Biol Regul. (2017) 64:39–48. doi: 10.1016/j.jbior.2016.12.001 28189457

[20] Hagiwara A Cornu M Cybulski N Polak P Betz C Trapani F . Hepatic mTORC2 activates glycolysis and lipogenesis through Akt, glucokinase, and SREBP1c. Cell Metab. (2012) 15:725–38. doi: 10.1016/j.cmet.2012.03.015 22521878

[21] Li M Wu X Pan Y Song M Yang X Xu J . mTORC2-AKT signaling to PFKFB2 activates glycolysis that enhances stemness and tumorigenicity of intestinal epithelial cells. FASEB J. (2024) 38:e23532. doi: 10.1096/fj.202301833RR 38451470

[22] Kim J Li J Wei J Lim SA . Regulatory T cell metabolism: a promising therapeutic target for cancer treatment? Immune Netw. (2025) 25:e13. doi: 10.4110/in.2025.25.e13 40078783 PMC11896657

[23] Almousa AA Morris M Fowler S Jones J Alcorn J . Elevation of serum pyruvate kinase M2 (PKM2) in IBD and its relationship to IBD indices. Clin Biochem. (2018) 53:19–24. doi: 10.1016/j.clinbiochem.2017.12.007 29273328

[24] Yılmaz İ Dolar ME Özpınar H . Effect of administering kefir on the changes in fecal microbiota and symptoms of inflammatory bowel disease: a randomized controlled trial. Turk J Gastroenterol. (2019) 30:242–53. doi: 10.5152/tjg.2018.18227 30662004 PMC6428516

[25] Danese S Levesque BG Feagan BG Jucov A Bhandari BR Pai RK . Randomised clinical trial: a phase 1b study of GB004, an oral HIF-1α stabiliser, for treatment of ulcerative colitis. Aliment Pharmacol Ther. (2022) 55:401–11. doi: 10.1111/apt.16753 35014040 PMC9305136

[26] Chen J Ruan X Sun Y Lu S Hu S Yuan S . Multi-omic insight into the molecular networks of mitochondrial dysfunction in the pathogenesis of inflammatory bowel disease. EBioMedicine. (2024) 99:104934. doi: 10.1016/j.ebiom.2023.104934 38103512 PMC10765009

[27] Fogt F Wellmann A Urbanski SJ Noffsinger A Poremba C Zimmerman RL . Glut-1 expression in dysplastic and regenerative lesions of the colon. Int J Mol Med. (2001) 7:615–9. doi: 10.3892/ijmm.7.6.615 11351274

[28] Weber-Stiehl S Taubenheim J Järke L Röcken C Schreiber S Aden K . Hexokinase 2 expression in apical enterocytes correlates with inflammation severity in patients with inflammatory bowel disease. BMC Med. (2024) 22:490. doi: 10.1186/s12916-024-03710-7 39444028 PMC11515617

[29] Barron CC Bilan PJ Tsakiridis T Tsiani E . Facilitative glucose transporters: implications for cancer detection, prognosis and treatment. Metabolism. (2016) 65:124–39. doi: 10.1016/j.metabol.2015.10.007 26773935

[30] Gao M Huang J Jiang X Yuan Y Pang H Luo S . Regulation of aerobic glycolysis to decelerate tumor proliferation by small molecule inhibitors targeting glucose transporters. Protein Cell. (2020) 11:446–51. doi: 10.1007/s13238-020-00725-7 32410006 PMC7251022

[31] Lv Q Wang K Qiao S Yang L Xin Y Dai Y . Norisoboldine, a natural AhR agonist, promotes Treg differentiation and attenuates colitis via targeting glycolysis and subsequent NAD+/SIRT1/SUV39H1/H3K9me3 signaling pathway. Cell Death Dis. (2018) 9:258. doi: 10.1038/s41419-018-0297-3 29449535 PMC5833367

[32] Chamarthy S Mekala JR . Functional importance of glucose transporters and chromatin epigenetic factors in Glioblastoma Multiforme (GBM): possible therapeutics. Metab Brain Dis. (2023) 38:1441–69. doi: 10.1007/s11011-023-01207-5 37093461

[33] Fontana F Giannitti G Marchesi S Limonta P . The PI3K/Akt pathway and glucose metabolism: a dangerous liaison in cancer. Int J Biol Sci. (2024) 20:3113–25. doi: 10.7150/ijbs.89942 38904014 PMC11186371

[34] Wang G Lai Y Chen X Li N Zhong C Yan Y . Hexokinase 2 promotes tumor development and progression. Am J Cancer Res. (2025) 15:4499–515. doi: 10.62347/ZYNN3077 41244129 PMC12616175

[35] Qian Y Zhu X Niu D Tang Q Jin C . Advancements in research on the role of the key glycolytic enzyme hexokinase 2 in the regulation of tumor immune evasion (Review). Oncol Lett. (2025) 30:593. doi: 10.3892/ol.2025.15339 41158312 PMC12557200

[36] Fang J Luo S Lu Z . HK2: Gatekeeping microglial activity by tuning glucose metabolism and mitochondrial functions. Mol Cell. (2023) 83:829–31. doi: 10.1016/j.molcel.2023.02.022 36931254

[37] Wu K Gong W Sun H Li W Chen L Duan Y . SMAD4 inhibits glycolysis in ovarian cancer through PI3K/AKT/HK2 signaling pathway by activating ARHGAP10. Cancer Rep (Hoboken). (2024) 7:e1976. doi: 10.1002/cnr2.1976 38230565 PMC10849991

[38] Mi Y Li Q Liu B Wang D Liu Z Wang T . Ubiquitous mitochondrial creatine kinase promotes the progression of gastric cancer through a JNK-MAPK/JUN/HK2 axis regulated glycolysis. Gastric Cancer. (2023) 26:69–81. doi: 10.1007/s10120-022-01340-7 36114400 PMC9813075

[39] Chen B Cai T Huang C Zang X Sun L Guo S . G6PD-NF-κB-HGF signal in gastric cancer-associated mesenchymal stem cells promotes the proliferation and metastasis of gastric cancer cells by upregulating the expression of HK2. Front Oncol. (2021) 11:648706. doi: 10.3389/fonc.2021.648706 33718248 PMC7952978

[40] Zhang X Sun Y Cheng S Yao Y Hua X Shi Y . CDK6 increases glycolysis and suppresses autophagy by mTORC1-HK2 pathway activation in cervical cancer cells. Cell Cycle. (2022) 21:984–1002. doi: 10.1080/15384101.2022.2039981 35167417 PMC9037534

[41] Kim JE He Q Chen Y Shi C Yu K . mTOR-targeted therapy: differential perturbation to mitochondrial membrane potential and permeability transition pore plays a role in therapeutic response. Biochem Biophys Res Commun. (2014) 447:184–91. doi: 10.1016/j.bbrc.2014.03.124 24704448

[42] Pastorino JG Hoek JB Shulga N . Activation of glycogen synthase kinase 3beta disrupts the binding of hexokinase II to mitochondria by phosphorylating voltage-dependent anion channel and potentiates chemotherapy-induced cytotoxicity. Cancer Res. (2005) 65:10545–54. doi: 10.1158/0008-5472.CAN-05-1925 16288047

[43] Wong N Ojo D Yan J Tang D . PKM2 contributes to cancer metabolism. Cancer Lett. (2015) 356:184–91. doi: 10.1016/j.canlet.2014.01.031 24508027

[44] Chen TJ Wu CH Hung MC Wang WC Kung HJ . Nuclear PKM2: a signal receiver, a gene programmer, and a metabolic modulator. J BioMed Sci. (2025) 32:75. doi: 10.1186/s12929-025-01170-6 40790192 PMC12341297

[45] Hitosugi T Kang S Vander Heiden MG Chung TW Elf S Lythgoe K . Tyrosine phosphorylation inhibits PKM2 to promote the Warburg effect and tumor growth. Sci Signal. (2009) 2:ra73. doi: 10.1126/scisignal.2000431 19920251 PMC2812789

[46] Lv L Li D Zhao D Lin R Chu Y Zhang H . Acetylation targets the M2 isoform of pyruvate kinase for degradation through chaperone-mediated autophagy and promotes tumor growth. Mol Cell. (2011) 42:719–30. doi: 10.1016/j.molcel.2011.04.025 21700219 PMC4879880

[47] Guo D Gu J Jiang H Ahmed A Zhang Z Gu Y . Inhibition of pyruvate kinase M2 by reactive oxygen species contributes to the development of pulmonary arterial hypertension. J Mol Cell Cardiol. (2016) 91:179–87. doi: 10.1016/j.yjmcc.2016.01.009 26774701

[48] Fan J Hitosugi T Chung TW Xie J Ge Q Gu TL . Tyrosine phosphorylation of lactate dehydrogenase A is important for NADH/NAD(+) redox homeostasis in cancer cells. Mol Cell Biol. (2011) 31:4938–50. doi: 10.1128/MCB.06120-11 21969607 PMC3233034

[49] Tudzarova S Colombo SL Stoeber K Carcamo S Williams GH Moncada S . Two ubiquitin ligases, APC/C-Cdh1 and SKP1-CUL1-F (SCF)-beta-TrCP, sequentially regulate glycolysis during the cell cycle. Proc Natl Acad Sci USA. (2011) 108:5278–83. doi: 10.1073/pnas.1102247108 21402913 PMC3069186

[50] Szwed A Kim E Jacinto E . Regulation and metabolic functions of mTORC1 and mTORC2. Physiol Rev. (2021) 101:1371–426. doi: 10.1152/physrev.00026.2020 33599151 PMC8424549

[51] Pollizzi KN Patel CH Sun IH Oh MH Waickman AT Wen J . mTORC1 and mTORC2 selectively regulate CD8^+^ T cell differentiation. J Clin Invest. (2015) 125:2090–108. doi: 10.1172/JCI77746 25893604 PMC4463194

[52] Hsu CY Huang JW Huang WR Chen IC Chen MS Liao TL . Oncolytic Avian Reovirus σA-modulated upregulation of the HIF-1α/C-myc/glut1 pathway to produce more energy in different cancer cell lines benefiting virus replication. Viruses. (2023) 15:523. doi: 10.3390/v15020523 36851737 PMC9961784

[53] Shi LZ Wang R Huang G Vogel P Neale G Green DR . HIF1alpha-dependent glycolytic pathway orchestrates a metabolic checkpoint for the differentiation of TH17 and Treg cells. J Exp Med. (2011) 208:1367–76. doi: 10.1084/jem.20110278 21708926 PMC3135370

[54] Delgoffe GM Pollizzi KN Waickman AT Heikamp E Meyers DJ Horton MR . The kinase mTOR regulates the differentiation of helper T cells through the selective activation of signaling by mTORC1 and mTORC2. Nat Immunol. (2011) 12:295–303. doi: 10.1038/ni.2005 21358638 PMC3077821

[55] Huang SC Smith AM Everts B Colonna M Pearce EL Schilling JD . Metabolic reprogramming mediated by the mTORC2-IRF4 signaling axis is essential for macrophage alternative activation. Immunity. (2016) 45:817–30. doi: 10.1016/j.immuni.2016.09.016 27760338 PMC5535820

[56] Heikamp EB Patel CH Collins S Waickman A Oh MH Sun IH . The AGC kinase SGK1 regulates TH1 and TH2 differentiation downstream of the mTORC2 complex. Nat Immunol. (2014) 15:457–64. doi: 10.1038/ni.2867 24705297 PMC4267697

[57] Fournier BM Parkos CA . The role of neutrophils during intestinal inflammation. Mucosal Immunol. (2012) 5:354–66. doi: 10.1038/mi.2012.24 22491176

[58] Williams IR Parkos CA . Colonic neutrophils in inflammatory bowel disease: double-edged swords of the innate immune system with protective and destructive capacity. Gastroenterology. (2007) 133:2049–52. doi: 10.1053/j.gastro.2007.10.031 18054577

[59] Gadaleta RM van Erpecum KJ Oldenburg B Willemsen EC Renooij W Murzilli S . Farnesoid X receptor activation inhibits inflammation and preserves the intestinal barrier in inflammatory bowel disease. Gut. (2011) 60:463–72. doi: 10.1136/gut.2010.212159 21242261

[60] Leblanc PO Bourgoin SG Poubelle PE Tessier PA Pelletier M . Metabolic regulation of neutrophil functions in homeostasis and diseases. J Leukoc Biol. (2024) 116:456–68. doi: 10.1093/jleuko/qiae025 38452242

[61] Kang D Li A Xie X Liu H Chen L Feng Z . Dysregulation of Farnesoid X Receptor on neutrophil homeostasis exacerbates intestinal inflammation via the mTORC1-glycolysis signaling pathway. MedComm (2020). (2026) 7:e70637. doi: 10.1002/mco2.70637 41664708 PMC12883035

[62] Zhang Z Dong L Jia A Chen X Yang Q Wang Y . Glucocorticoids promote the onset of acute experimental colitis and cancer by upregulating mTOR signaling in intestinal epithelial cells. Cancers (Basel). (2020) 12:945. doi: 10.3390/cancers12040945 32290362 PMC7254274

[63] Cheng J Zhang Y Ma L Du W Zhang Q . Macrophage-derived extracellular vesicles-coated palladium nanoformulations modulate inflammatory and immune homeostasis for targeting therapy of ulcerative colitis. Adv Sci (Weinh). (2023) 10:e2304002. doi: 10.1002/advs.202304002 37807805 PMC10667822

[64] Zhao H Hu Z . Activation of IL-17/CEBPB/PFKFB3 triggers energy metabolic switching and goblet cell differentiation in ulcerative colitis. Biochem Pharmacol. (2026) 245:117654. doi: 10.1016/j.bcp.2025.117654 41418940

[65] Li S Zhu J Song J Yang L Gong Y Dai Y . Aryl hydrocarbon receptor impairs HK2-controlled flux of the hexosamine biosynthesis pathway to suppress NETosis in an N-glycosylation-dependent manner. J Adv Res. (2026) 82:1069–82. doi: 10.1016/j.jare.2025.06.078 40582563 PMC13000937

[66] Na YR Stakenborg M Seok SH Matteoli G . Macrophages in intestinal inflammation and resolution: a potential therapeutic target in IBD. Nat Rev Gastroenterol Hepatol. (2019) 16:531–43. doi: 10.1038/s41575-019-0172-4 31312042

[67] Mowat AM Agace WW . Regional specialization within the intestinal immune system. Nat Rev Immunol. (2014) 14:667–85. doi: 10.1038/nri3738 25234148

[68] Covarrubias AJ Aksoylar HI Horng T . Control of macrophage metabolism and activation by mTOR and Akt signaling. Semin Immunol. (2015) 27:286–96. doi: 10.1016/j.smim.2015.08.001 26360589 PMC4682888

[69] Zaiatz Bittencourt V Jones F Doherty G Ryan EJ . Targeting immune cell metabolism in the treatment of inflammatory bowel disease. Inflammation Bowel Dis. (2021) 27:1684–93. doi: 10.1093/ibd/izab024 33693743 PMC8522790

[70] Masoud GN Li W . HIF-1α pathway: role, regulation and intervention for cancer therapy. Acta Pharm Sin B. (2015) 5:378–89. doi: 10.1016/j.apsb.2015.05.007 26579469 PMC4629436

[71] Collins SL Oh MH Sun IH Chan-Li Y Zhao L Powell JD . mTORC1 signaling regulates proinflammatory macrophage function and metabolism. J Immunol. (2021) 207:913–22. doi: 10.4049/jimmunol.2100230 34290107

[72] Wu MM Wang QM Huang BY Mai CT Wang CL Wang TT . Dioscin ameliorates murine ulcerative colitis by regulating macrophage polarization. Pharmacol Res. (2021) 172:105796. doi: 10.1016/j.phrs.2021.105796 34343656

[73] Li YZ Chao R Qu SL Huang L Zhang C . ZNF667 suppressed LPS-induced macrophages inflammation through mTOR-dependent aerobic glycolysis regulation. Curr Pharm Des. (2023) 29:1361–9. doi: 10.2174/1381612829666230530143129 37259213

[74] Zhuang H Lv Q Zhong C Cui Y He L Zhang C . Tiliroside ameliorates ulcerative colitis by restoring the M1/M2 macrophage balance via the HIF-1α/glycolysis pathway. Front Immunol. (2021) 12:649463. doi: 10.3389/fimmu.2021.649463 33868286 PMC8044352

[75] Li J Li M Ye K Jiang Q Wang M Wen X . Chemical profile of Xian-He-Cao-Chang-Yan formula and its effects on ulcerative colitis. J Ethnopharmacol. (2021) 267:113517. doi: 10.1016/j.jep.2020.113517 33164773

[76] Akanyibah FA He C Wang X Wang B Mao F . The role of plant-based dietary compounds in gut microbiota modulation in inflammatory bowel disease. Front Nutr. (2025) 12:1606289. doi: 10.3389/fnut.2025.1606289 40521353 PMC12163340

[77] Vivier E Artis D Colonna M Diefenbach A Di Santo JP Eberl G . Innate lymphoid cells: 10 years on. Cell. (2018) 174:1054–66. doi: 10.1016/j.cell.2018.07.017 30142344

[78] Sonnenberg GF Hepworth MR . Functional interactions between innate lymphoid cells and adaptive immunity. Nat Rev Immunol. (2019) 19:599–613. doi: 10.1038/s41577-019-0194-8 31350531 PMC6982279

[79] Geremia A Arancibia-Cárcamo CV Fleming MP Rust N Singh B Mortensen NJ . IL-23-responsive innate lymphoid cells are increased in inflammatory bowel disease. J Exp Med. (2011) 208:1127–33. doi: 10.1084/jem.20101712 21576383 PMC3173242

[80] Zenewicz LA Yancopoulos GD Valenzuela DM Murphy AJ Stevens S Flavell RA . Innate and adaptive interleukin-22 protects mice from inflammatory bowel disease. Immunity. (2008) 29:947–57. doi: 10.1016/j.immuni.2008.11.003 19100701 PMC3269819

[81] Serafini N Jarade A Surace L Goncalves P Sismeiro O Varet H . Trained ILC3 responses promote intestinal defense. Science. (2022) 375:859–63. doi: 10.1126/science.aaz8777 35201883 PMC10351749

[82] Di Luccia B Gilfillan S Cella M Colonna M Huang SC . ILC3s integrate glycolysis and mitochondrial production of reactive oxygen species to fulfill activation demands. J Exp Med. (2019) 216:2231–41. doi: 10.1084/jem.20180549 31296736 PMC6781001

[83] Sepahi A Liu Q Friesen L Kim CH . Dietary fiber metabolites regulate innate lymphoid cell responses. Mucosal Immunol. (2021) 14:317–30. doi: 10.1038/s41385-020-0312-8 32541842 PMC7736174

[84] Vonarbourg C Mortha A Bui VL Hernandez PP Kiss EA Hoyler T . Regulated expression of nuclear receptor RORγt confers distinct functional fates to NK cell receptor-expressing RORγt(+) innate lymphocytes. Immunity. (2010) 33:736–51. doi: 10.1016/j.immuni.2010.10.017 21093318 PMC3042726

[85] Zeng B Shi S Ashworth G Dong C Liu J Xing F . ILC3 function as a double-edged sword in inflammatory bowel diseases. Cell Death Dis. (2019) 10:315. doi: 10.1038/s41419-019-1540-2 30962426 PMC6453898

[86] Yan R Jia D Qi Y Wang Q Chen S . Intestinal tissue-resident memory T cells: characteristics, spatial heterogeneity, age-related dynamics, and roles in disease regulation. J Adv Res. (2026) 79:535–47. doi: 10.1016/j.jare.2025.03.021 40096943 PMC12766223

[87] Gomez-Bris R Saez A Herrero-Fernandez B Rius C Sanchez-Martinez H Gonzalez-Granado JM . CD4 T-cell subsets and the pathophysiology of inflammatory bowel disease. Int J Mol Sci. (2023) 24:2696. doi: 10.3390/ijms24032696 36769019 PMC9916759

[88] Xia X Huang Z Xu C Fu H Wang S Tian J . Regulation of intestinal tissue-resident memory T cells: a potential target for inflammatory bowel disease. Cell Commun Signal. (2024) 22:610. doi: 10.1186/s12964-024-01984-1 39695803 PMC11658174

[89] Rooks MG Garrett WS . Gut microbiota, metabolites and host immunity. Nat Rev Immunol. (2016) 16:341–52. doi: 10.1038/nri.2016.42 27231050 PMC5541232

[90] Parthasarathy A Li T Edelblum KL . Crosstalk between the microbiota and intestinal γδ T cell compartments in health and IBD. Gut Microbes. (2026) 18:2604908. doi: 10.1080/19490976.2025.2604908 41459728 PMC12758196

[91] Xing Y Wang M Yuan Y Hu J Wang Z Sun Z . Gut microbiota-derived butyrate mediates the anticolitic effect of indigo supplementation through regulating CD4+ T cell differentiation. Imeta. (2025) 4:e70040. doi: 10.1002/imt2.70040 40469515 PMC12130576

[92] Lee C Park YW Park MH Lee YJ Rhee I . Regulatory T cells and their role in inflammatory bowel disease: molecular targets, therapeutic strategies and translational advances. Biochem Pharmacol. (2025) 239:117087. doi: 10.1016/j.bcp.2025.117087 40571217

[93] Xu Y Cai R Zhao Z Zhou L Zhou Q Hassan S . Thiomyristoyl ameliorates colitis by blocking the differentiation of Th17 cells and inhibiting SIRT2-induced metabolic reprogramming. Int Immunopharmacol. (2021) 90:107212. doi: 10.1016/j.intimp.2020.107212 33310666

[94] Ma Z Wang Z Cao J Dong Y Chen Y . Regulatory roles of intestinal CD4+ T cells in inflammation and their modulation by the intestinal microbiota. Gut Microbes. (2025) 17:2560019. doi: 10.1080/19490976.2025.2560019 40963293 PMC12452487

[95] Hu J Wang W Wang M Wu C Jiao Y Li Y . Immunological pathogenesis of inflammatory bowel disease: focus on tissue resident memory T cells. Front Immunol. (2025) 16:1591584. doi: 10.3389/fimmu.2025.1591584 40529369 PMC12170311

[96] Hu Y Zhao Q Dai H Wu Y Tang X Zhang N . Metabolic reprogramming as a therapeutic target for modulating the Th17/Treg balance in autoimmune diseases: a comprehensive review. Front Immunol. (2025) 16:1687755. doi: 10.3389/fimmu.2025.1687755 41476956 PMC12747992

[97] Wang Z Xu Z Zong M Fan L . Metabolic regulation of Th17 and Treg cell balance by the mTOR signaling. Metabol Open. (2025) 26:100369. doi: 10.1016/j.metop.2025.100369 40476179 PMC12140031

[98] Brescia C Audia S Pugliano A Scaglione F Iuliano R Trapasso F . Metabolic drives affecting Th17/Treg gene expression changes and differentiation: impact on immune-microenvironment regulation. APMIS. (2024) 132:1026–45. doi: 10.1111/apm.13378 38239016

[99] Shan J Jin H Xu Y . T cell metabolism: a new perspective on Th17/Treg cell imbalance in systemic lupus erythematosus. Front Immunol. (2020) 11:1027. doi: 10.3389/fimmu.2020.01027 32528480 PMC7257669

[100] Wang Y Li M Zha A . mTOR promotes an inflammatory response through the HIF1 signaling pathway in ulcerative colitis. Int Immunopharmacol. (2024) 134:112217. doi: 10.1016/j.intimp.2024.112217 38718658

[101] Liu M Luo J Huang J Zhu X Liu D Long J . Mechanisms of astragalus polysaccharide alleviated experimental colitis involved mTreg cells and the mTOR/HIF-1α pathway. J Nutr Biochem. (2025) 145:110010. doi: 10.1016/j.jnutbio.2025.110010 40614837

[102] Yang W Liu H Xu L Yu T Zhao X Yao S . GPR120 inhibits colitis through regulation of CD4+ T cell interleukin 10 production. Gastroenterology. (2022) 162:150–65. doi: 10.1053/j.gastro.2021.09.018 34536451 PMC8678294

[103] Shen H Shi LZ . Metabolic regulation of TH17 cells. Mol Immunol. (2019) 109:81–7. doi: 10.1016/j.molimm.2019.03.005 30903829 PMC7059830

[104] Piedra-Quintero ZL Serrano C Villegas-Sepúlveda N Maravillas-Montero JL Romero-Ramírez S Shibayama M . Myosin 1F regulates M1-polarization by stimulating intercellular adhesion in macrophages. Front Immunol. (2019) 9:3118. doi: 10.3389/fimmu.2018.03118 30687322 PMC6335276

[105] Li H Fan C Feng C Wu Y Lu H He P . Inhibition of phosphodiesterase-4 attenuates murine ulcerative colitis through interference with mucosal immunity. Br J Pharmacol. (2019) 176:2209–26. doi: 10.1111/bph.14667 30883697 PMC6555859

[106] Qu S Shen Y Wang M Wang X Yang Y . Suppression of miR-21 and miR-155 of macrophage by cinnamaldehyde ameliorates ulcerative colitis. Int Immunopharmacol. (2019) 67:22–34. doi: 10.1016/j.intimp.2018.11.045 30530166

[107] Arosa L Camba-Gómez M Lorenzo-Martín LF Clavaín L López M Conde-Aranda J . RNA expression of MMP12 is strongly associated with inflammatory bowel disease and is regulated by metabolic pathways in RAW 264.7 macrophages. Int J Mol Sci. (2024) 25:3167. doi: 10.3390/ijms25063167 38542140 PMC10970096

[108] Ignacio A Cipelli M Takiishi T Favero Aguiar C Fernandes Terra F Ghirotto B . Lack of mTORC2 signaling in CD11c+ myeloid cells inhibits their migration and ameliorates experimental colitis. J Leukoc Biol. (2024) 116:779–92. doi: 10.1093/jleuko/qiae084 38652699

[109] Wu X Zhang Q Peng L Tian Z Gou G Zuo W . Colon-targeted piperine-glycyrrhizic acid nanocrystals for ulcerative colitis synergetic therapy via macrophage polarization. J Mater Chem B. (2024) 12:1604–16. doi: 10.1039/d3tb02312e 38269414

[110] Zhang H Zhao X Gao Y Shi Y Wei L Li J . D-Mannose promotes recovery from experimental colitis by inducing AMPK phosphorylation to stimulate epithelial repair. Food Funct. (2024) 15:625–46. doi: 10.1039/d3fo03146b 38099724

[111] Kasai S Karmacharya A Mukai Y Sato S . Bangle (Zingiber purpureum Rosc.) extract ameliorates colonic inflammation and upregulates autophagy via the modulation of the AMPK/mTOR/NFκB pathway in a mouse colitis model. Mol Nutr Food Res. (2025) 69:e70034. doi: 10.1002/mnfr.70034 40177841

[112] Yaxian L Xiaodong W Futao M Huizhen W Chuansheng W Mengdi M . SAMHD1 deficiency disrupts macrophage autophagy-lysosomal homeostasis and promotes inflammation via the mTOR-MITF-CTSD axis in ulcerative colitis. Int J Biol Macromol. (2025) 327:147188. doi: 10.1002/mnfr.70034 40886983

[113] He L Zhong Z Liu F Wen S . Berberine alleviates DSS-induced colitis by modulating macrophage phenotype via PPAR-γ/ mTOR/HIF-1α signaling pathway. J Ethnopharmacol. (2026) 362:121350. doi: 10.1016/j.jep.2026.121350 41679358

[114] Li S Yuan G Chen C Lu J Zhang X . Wenyang decoction ameliorates DSS-induced ulcerative colitis by regulating macrophage polarization and the PI3K/AKT/mTOR/HIF-1α signaling pathway. Mediators Inflammation. (2026) 2026:7737168. doi: 10.1155/mi/7737168 41800106 PMC12966625

[115] Qu L Lin X Liu C Ke C Zhou Z Xu K . Atractylodin attenuates dextran sulfate sodium-induced colitis by alleviating gut microbiota dysbiosis and inhibiting inflammatory response through the MAPK pathway. Front Pharmacol. (2021) 12:665376. doi: 10.3389/fphar.2021.665376 34335244 PMC8320761

[116] Huang B Wang Q Jiang L Lu S Li C Xu C . Shikonin ameliorated mice colitis by inhibiting dimerization and tetramerization of PKM2 in macrophages. Front Pharmacol. (2022) 13:926945. doi: 10.3389/fphar.2022.926945 36059938 PMC9428403

[117] Yuan Y Ni S Zhuge A Li L Li B . Adipose-derived mesenchymal stem cells reprogram M1 macrophage metabolism via PHD2/HIF-1α pathway in colitis mice. Front Immunol. (2022) 13:859806. doi: 10.3389/fimmu.2022.859806 35757749 PMC9226317

[118] Wang F Luo L Wu Z Wan L Li F Wen Z . HMGB1 modulates macrophage metabolism and polarization in ulcerative colitis by inhibiting Cpt1a expression. Front Biosci (Landmark Ed). (2024) 29:387. doi: 10.31083/j.fbl2911387 39614449

[119] Fang Y Chen Y Yu X Zhang J Zhu W Mou C . Rg1-preconditioned adipose-derived mesenchymal stromal cells alleviate colitis via exosome-mediated inhibition of macrophage glycolysis through RAS signaling. Stem Cell Res Ther. (2025) 17:68. doi: 10.1186/s13287-025-04822-4 41316493 PMC12870250

[120] Guo Y Shen A Han K Wang R Wang Q Wei J . Potentilla anserina L. flavonoids ameliorate ulcerative colitis by modulating oxidative stress, intestinal microbiota dysbiosis, and inflammatory responses. 3 Biotech. (2025) 15:245. doi: 10.1007/s13205-025-04410-6 40636944 PMC12234962

[121] Han Y Xun J Li T Jiang X Liu B Hu Z . FATS alleviates ulcerative colitis by inhibiting M1 macrophage polarization and aerobic glycolysis through promoting the ubiquitination-mediated degradation of HIF-1α. Biochem Pharmacol. (2025) 240:117053. doi: 10.1016/j.bcp.2025.117053 40543763

[122] Li J Zou P Xiao R Wang Y . Indole-3-propionic acid alleviates DSS-induced colitis in mice through macrophage glycolipid metabolism. Int Immunopharmacol. (2025) 152:114388. doi: 10.1016/j.intimp.2025.114388 40086057

[123] Ma C Chen K Li L Jiang M Zeng Z Yin F . Epstein-Barr virus infection exacerbates ulcerative colitis by driving macrophage pyroptosis via the upregulation of glycolysis. Precis Clin Med. (2025) 8:pbaf002. doi: 10.1093/pcmedi/pbaf002 40041420 PMC11878796

[124] Pan X Ren Z Liang W Dong X Li J Wang L . Thiamine deficiency aggravates experimental colitis in mice by promoting glycolytic reprogramming in macrophages. Br J Pharmacol. (2025) 182:1897–911. doi: 10.1111/bph.17435 39890689

[125] Zhang J Lin X Song H Xie Y Hou S Huang S . Dendrobium officinale polysaccharide inhibits M1 macrophage polarization via activating SENP1-SIRT3 signaling and alleviates ulcerative colitis. Food Res Int. (2025) 221:117582. doi: 10.1016/j.foodres.2025.117582 41185333

[126] Yan S Hui Y Li J Xu X Li Q Wei H . Glutamine relieves oxidative stress through PI3K/Akt signaling pathway in DSS-induced ulcerative colitis mice. Iran J Basic Med Sci. (2020) 23:1124–9. doi: 10.22038/ijbms.2020.39815.9436 32963733 PMC7491493

[127] Zhu F Jing D Zhou H Hu Z Wang Y Jin G . Blockade of Syk modulates neutrophil immune-responses via the mTOR/RUBCNL-dependent autophagy pathway to alleviate intestinal inflammation in ulcerative colitis. Precis Clin Med. (2023) 6:pbad025. doi: 10.1093/pcmedi/pbad025 37941642 PMC10628969

[128] Chen C Sun B Chen K Bao H Tao Y Zhou J . Atractylenolide-I restore intestinal barrier function by targeting the S100A9/AMPK/mTOR signaling pathway. Front Pharmacol. (2025) 16:1530109. doi: 10.3389/fphar.2025.1530109 40196359 PMC11973269

[129] Yao J Wei C Wang JY Zhang R Li YX Wang LS . Effect of resveratrol on Treg/Th17 signaling and ulcerative colitis treatment in mice. World J Gastroenterol. (2015) 21:6572–81. doi: 10.3748/wjg.v21.i21.6572 26074695 PMC4458767

[130] Zhang D Wei C Yao J Cai X Wang L . Interleukin-10 gene-carrying bifidobacteria ameliorate murine ulcerative colitis by regulating regulatory T cell/T helper 17 cell pathway. Exp Biol Med (Maywood). (2015) 240:1622–9. doi: 10.1177/1535370215584901 25956685 PMC4935350

[131] Kulkarni N Meitei HT Sonar SA Sharma PK Mujeeb VR Srivastava S . CCR6 signaling inhibits suppressor function of induced-Treg during gut inflammation. J Autoimmun. (2018) 88:121–30. doi: 10.1016/j.jaut.2017.10.013 29126851

[132] Lin X Sun Q Zhou L He M Dong X Lai M . Colonic epithelial mTORC1 promotes ulcerative colitis through COX-2-mediated Th17 responses. Mucosal Immunol. (2018) 11:1663–73. doi: 10.1038/s41385-018-0018-3 30082707

[133] Youssef ME Abd El-Fattah EE Abdelhamid AM Eissa H El-Ahwany E Amin NA . Interference with the AMPKα/mTOR/NLRP3 signaling and the IL-23/IL-17 axis effectively protects against the dextran sulfate sodium intoxication in rats: a new paradigm in empagliflozin and metformin reprofiling for the management of ulcerative colitis. Front Pharmacol. (2021) 12:719984. doi: 10.3389/fphar.2021.719984 34489707 PMC8417441

[134] He H Chen Q Fan H Leng XY Zhu F Gao F . Extracellular vesicles produced by bone marrow mesenchymal stem cells overexpressing programmed death-ligand 1 ameliorate dextran sodium sulfate-induced ulcerative colitis in rats by regulating Th17/Treg cell balance through PTEN/PI3K/AKT/mTOR axis. J Gastroenterol Hepatol. (2022) 37:2243–54. doi: 10.1111/jgh.15987 36044618 PMC10087423

[135] Yang YX Yuan Y Xia B . Cinnamtannin D1 ameliorates DSS-induced colitis by preventing Th17/Treg imbalance through activation of the AMPK/mTOR pathway. Allergol Immunopathol (Madr). (2022) 50:153–61. doi: 10.15586/aei.v50i5.654 36086976

[136] Li M Wang T Yuan M . Molecular mechanism of butyrate modulating Treg/Th17 balance in UC through cAMP-PKA/mTOR axis. Immunol Invest. (2025) 54:1501–23. doi: 10.1080/08820139.2025.2556790 40916037

[137] Wang G Han L Sun H Sun T Wu B Cheng X . Artesunate promotes intestinal epithelial barrier repair by facilitating HMGCS2-dependent ketogenesis in experimental ulcerative colitis model mice. Int Immunopharmacol. (2025) 162:115152. doi: 10.1016/j.intimp.2025.115152 40614600

[138] Kaur H Moreau R . Role of mTORC1 in intestinal epithelial repair and tumorigenesis. Cell Mol Life Sci. (2019) 76:2525–46. doi: 10.1016/j.intimp.2025.115152 30944973 PMC11105546

[139] Lv Q Xing Y Dong D Hu Y Chen Q Zhai L . Costunolide ameliorates colitis via specific inhibition of HIF1α/glycolysis-mediated Th17 differentiation. Int Immunopharmacol. (2021) 97:107688. doi: 10.1016/j.intimp.2021.107688 33932695

[140] Liu S Yan W Lv Q Yang L Miao Y Hu Y . 3, 3'-diindolylmethane, a natural aryl hydrocarbon receptor agonist, alleviates ulcerative colitis by enhancing "glycolysis-lactate-STAT3″ and TIP60 signals-mediated Treg differentiation. Mol Immunol. (2023) 163:147–62. doi: 10.1016/j.molimm.2023.09.009 37793204

[141] Wang P Yang H Lin W Zhou J Liu Y Ma L . Discovery of novel sesquiterpene lactone derivatives as potent PKM2 activators for the treatment of ulcerative colitis. J Med Chem. (2023) 66:5500–23. doi: 10.1021/acs.jmedchem.2c01856 37017305

[142] Yang L Zhu JC Li SJ Zeng X Xue XR Dai Y . HSP90β shapes the fate of Th17 cells with the help of glycolysis-controlled methylation modification. Br J Pharmacol. (2024) 181:3886–907. doi: 10.1111/bph.16432 38881036

[143] Huo L Chen Q Jia S Zhang Y Wang L Li X . Gut microbiome promotes succinate-induced ulcerative colitis by enhancing glycolysis through SUCNR1/NF-κB signaling pathway. Am J Physiol Cell Physiol. (2025) 329:C440–54. doi: 10.1152/ajpcell.00411.2025 40549551

[144] Yang GM Jiang HY Ren SH Xu YN Wang HD Shao B . Oral microcapsules encapsulating endometrial regenerative cell-derived exosomes promote intestinal epithelial barrier repair and ameliorate experimental colitis. Int J Nanomedicine. (2026) 21:540230. doi: 10.2147/IJN.S540230 41907368 PMC13022928

[145] Sampson LL Davis AK Grogg MW Zheng Y . mTOR disruption causes intestinal epithelial cell defects and intestinal atrophy postinjury in mice. FASEB J. (2016) 30:1263–75. doi: 10.1096/fj.15-278606 26631481 PMC4750410

[146] Rodríguez-Colman MJ Schewe M Meerlo M Stigter E Gerrits J Pras-Raves M . Interplay between metabolic identities in the intestinal crypt supports stem cell function. Nature. (2017) 543:424–7. doi: 10.1038/nature21673 28273069

[147] Yang Q Zhang P Han L Shi P Zhao Z Cui D . Mitochondrial-related genes PDK2, CHDH, and ALDH5A1 served as a diagnostic signature and correlated with immune cell infiltration in ulcerative colitis. Aging (Albany NY). (2024) 16:3803–22. doi: 10.18632/aging.205561 38376420 PMC10929806

[148] Bhonde MR Gupte RD Dadarkar SD Jadhav MG Tannu AA Bhatt P . A novel mTOR inhibitor is efficacious in a murine model of colitis. Am J Physiol Gastrointest Liver Physiol. (2008) 295:G1237–1245. doi: 10.1152/ajpgi.90537.2008 18927209

[149] Kang SB Yoo HS Jeon SH Song CW Lee NR Kim NJ . Identification of 3",4",5"-trihydroxyflavone as an mammalian target of rapamycin inhibitor and its suppressive effects on dextran sulfate sodium-induced ulcerative colitis. Int Immunopharmacol. (2020) 84:106524. doi: 10.1016/j.intimp.2020.106524 32334388

[150] Zhang F Wang W Niu J Yang G Luo J Lan D . Heat-shock transcription factor 2 promotes sodium butyrate-induced autophagy by inhibiting mTOR in ulcerative colitis. Exp Cell Res. (2020) 388:111820. doi: 10.1016/j.yexcr.2020.111820 31923427

[151] Zhang SL Li ZY Wang DS Xu TY Fan MB Cheng MH . Aggravated ulcerative colitis caused by intestinal Metrnl deficiency is associated with reduced autophagy in epithelial cells. Acta Pharmacol Sin. (2020) 41:763–70. doi: 10.1038/s41401-019-0343-4 31949292 PMC7471395

[152] Zhou J Wang J Li D Zhang Z Wang C Zhang X . An inulin-type fructan CP-A from Codonopsis pilosula alleviates TNBS-induced ulcerative colitis based on serum-untargeted metabolomics. Am J Physiol Gastrointest Liver Physiol. (2024) 326:G216–27. doi: 10.1152/ajpgi.00214.2023 38193197

[153] Zhang Y Lai Z Hu X Wu M Chen S Su L . Palmatine ameliorates intestinal epithelial barrier injury in ulcerative colitis via targeting enolase 3. Int Immunopharmacol. (2025) 162:115110. doi: 10.1016/j.intimp.2025.115110 40543101

[154] Li D Feng Y Tian M Ji J Hu X Chen F . Gut microbiota-derived inosine from dietary barley leaf supplementation attenuates colitis through PPARγ signaling activation. Microbiome. (2021) 9:83. doi: 10.1186/s40168-021-01028-7 33820558 PMC8022418

[155] Xu Y Ou J Zhang C Chen J Chen J Li A . Rapamycin promotes the intestinal barrier repair in ulcerative colitis via the mTOR/PBLD/AMOT signaling pathway. Biochim Biophys Acta Mol Basis Dis. (2024) 1870:167287. doi: 10.1016/j.bbadis.2024.167287 38862095

[156] Lan A Guerbette T Andriamihaja M Magnin B Bordet M Ferron PJ . Mitochondrial remodeling and energy metabolism adaptations in colonic crypts during spontaneous epithelial repair after colitis induction in mice. Free Radic Biol Med. (2023) 205:224–33. doi: 10.1016/j.freeradbiomed.2023.06.007 37315703

[157] Hall CHT Lee JS Murphy EM Gerich ME Dran R Glover LE . Creatine transporter, reduced in colon tissues from patients with inflammatory bowel diseases, regulates energy balance in intestinal epithelial cells, epithelial integrity, and barrier function. Gastroenterology. (2020) 159:984–98. doi: 10.1053/j.gastro.2020.05.033 32433978 PMC7891846

[158] Zhu J Wu Y Zhang L Bai B Han W Wang H . Epithelial Piezo1 deletion ameliorates intestinal barrier damage by regulating ferroptosis in ulcerative colitis. Free Radic Biol Med. (2024) 224:272–86. doi: 10.1016/j.freeradbiomed.2024.08.039 39216559

[159] Liu T Ou G Wu J Wang S Wang H Wu Z . Pingwei Powder alleviates high-fat diet-induced colonic inflammation by modulating microbial metabolites SCFAs. Front Cell Infect Microbiol. (2025) 15:1628488. doi: 10.3389/fcimb.2025.1628488 41036224 PMC12479493

[160] Xie B Wang M Xiao Y Zhang X Liu M Miao J . Tryptophan metabolic gatekeeping in epithelial repair: GPR35-KLF5 circuitry decodes mucosal damage signals for repair programming. Cell Death Dis. (2026) 17:25. doi: 10.1038/s41419-025-08237-0 41513630 PMC12789062

[161] Allaire JM Crowley SM Law HT Chang SY Ko HJ Vallance BA . The intestinal epithelium: Central coordinator of mucosal immunity. Trends Immunol. (2018) 39:677–96. doi: 10.1016/j.it.2018.04.002 29716793

[162] Biton M Haber AL Rogel N Burgin G Beyaz S Schnell A . T helper cell cytokines modulate intestinal stem cell renewal and differentiation. Cell. (2018) 175:1307–20.e22. doi: 10.1016/j.cell.2018.10.008 30392957 PMC6239889

[163] De Schepper S Verheijden S Aguilera-Lizarraga J Viola MF Boesmans W Stakenborg N . Self-maintaining gut macrophages are essential for intestinal homeostasis. Cell. (2019) 176:676. doi: 10.1016/j.cell.2018.07.048 30682373

[164] Muller PA Koscsó B Rajani GM Stevanovic K Berres ML Hashimoto D . Crosstalk between muscularis macrophages and enteric neurons regulates gastrointestinal motility. Cell. (2014) 158:1210. doi: 10.1016/j.cell.2014.08.002 28917294

[165] Sehgal A Donaldson DS Pridans C Sauter KA Hume DA Mabbott NA . The role of CSF1R-dependent macrophages in control of the intestinal stem-cell niche. Nat Commun. (2018) 9:1272. doi: 10.1038/s41467-018-03638-6 29593242 PMC5871851

[166] Kaur H Erickson A Moreau R . Divergent regulation of inflammatory cytokines by mTORC1 in THP-1-derived macrophages and intestinal epithelial Caco-2 cells. Life Sci. (2021) 284:119920. doi: 10.1016/j.lfs.2021.119920 34478760

[167] Chancharoenthana W Kamolratanakul S Udompornpitak K Wannigama DL Schultz MJ Leelahavanichkul A . Alcohol-induced gut permeability defect through dysbiosis and enterocytic mitochondrial interference causing pro-inflammatory macrophages in a dose dependent manner. Sci Rep. (2025) 15:14710. doi: 10.1038/s41598-025-97593-0 40289168 PMC12034794

[168] Heuberger CE Janney A Ilott N Bertocchi A Pott S Gu Y . MHC class II antigen presentation by intestinal epithelial cells fine-tunes bacteria-reactive CD4 T-cell responses. Mucosal Immunol. (2024) 17:416–30. doi: 10.1016/j.mucimm.2023.05.001 37209960

[169] Backert I Koralov SB Wirtz S Kitowski V Billmeier U Martini E . STAT3 activation in Th17 and Th22 cells controls IL-22-mediated epithelial host defense during infectious colitis. J Immunol. (2014) 193:3779–91. doi: 10.4049/jimmunol.1303076 25187663

[170] Luo Y Zhang Z Ren J Dou C Wen J Yang Y . SARS-Cov-2 spike induces intestinal barrier dysfunction through the interaction between CEACAM5 and Galectin-9. Front Immunol. (2024) 15:1303356. doi: 10.3389/fimmu.2024.1303356 38686388 PMC11056506

[171] Parada Venegas D De la Fuente MK Landskron G González MJ Quera R Dijkstra G . Short chain fatty acids (SCFAs)-mediated gut epithelial and immune regulation and its relevance for inflammatory bowel diseases. Front Immunol. (2019) 10:277. doi: 10.3389/fimmu.2019.00277 30915065 PMC6421268

[172] Stadnyk AW . Intestinal epithelial cells as a source of inflammatory cytokines and chemokines. Can J Gastroenterol. (2002) 16:241–6. doi: 10.1155/2002/941087 11981577

[173] Turner JR . Intestinal mucosal barrier function in health and disease. Nat Rev Immunol. (2009) 9:799–807. doi: 10.1038/nri2653 19855405

[174] Cerutti A Rescigno M . The biology of intestinal immunoglobulin A responses. Immunity. (2008) 28:740–50. doi: 10.1016/j.immuni.2008.05.001 18549797 PMC3057455

[175] Tezuka H Abe Y Asano J Sato T Liu J Iwata M . Prominent role for plasmacytoid dendritic cells in mucosal T cell-independent IgA induction. Immunity. (2011) 34:247–57. doi: 10.1016/j.immuni.2011.02.002 21333555

[176] Pelaseyed T Bergström JH Gustafsson JK Ermund A Birchenough GM Schütte A . The mucus and mucins of the goblet cells and enterocytes provide the first defense line of the gastrointestinal tract and interact with the immune system. Immunol Rev. (2014) 260:8–20. doi: 10.1111/imr.12182 24942678 PMC4281373

[177] Bregman E Kirsner JB . Amino acids of colon and rectum. Possible involvement of diaminopimelic acid of intestinal bacteria in antigenicity of ulcerative colitis colon. Proc Soc Exp Biol Med. (1965) 118:727–31. doi: 10.3181/00379727-118-29952 14264542

[178] Lin J Zhang X Zhao Z Welker NC Li Y Liu Y . Novel microRNA signature to differentiate ulcerative colitis from Crohn disease: A genome-wide study using next generation sequencing. Microrna. (2016) 5:222–9. doi: 10.2174/2211536605666161117113031 27855604

[179] Perl A . mTOR activation is a biomarker and a central pathway to autoimmune disorders, cancer, obesity, and aging. Ann N Y Acad Sci. (2015) 1346:33–44. doi: 10.1111/nyas.12756 25907074 PMC4480196

[180] Liu GY Sabatini DM . mTOR at the nexus of nutrition, growth, ageing and disease. Nat Rev Mol Cell Biol. (2020) 21:183–203. doi: 10.1038/s41580-019-0199-y 31937935 PMC7102936

[181] Marafie SK Al-Mulla F Abubaker J . mTOR: Its critical role in metabolic diseases, cancer, and the aging process. Int J Mol Sci. (2024) 25:6141. doi: 10.3390/ijms25116141 38892329 PMC11173325

[182] Yang K Chi H . mTOR and metabolic pathways in T cell quiescence and functional activation. Semin Immunol. (2012) 24:421–8. doi: 10.1016/j.smim.2012.12.004 23375549 PMC3855395

[183] Cai X Li H Wang M Chu E Wei N Lin J . mTOR participates in the formation, maintenance, and function of memory CD8+T cells regulated by glycometabolism. Biochem Pharmacol. (2022) 204:115197. doi: 10.1016/j.bcp.2022.115197 35926651

[184] Kaminski H Marseres G Yared N Nokin MJ Pitard V Zouine A . mTOR inhibitors prevent CMV infection through the restoration of functional αβ and γδ T cells in kidney transplantation. J Am Soc Nephrol. (2022) 33:121–37. doi: 10.1681/ASN.2020121753 34725108 PMC8763189

[185] Zurlo M Nicoli F Proietto D Dallan B Zuccato C Cosenza LC . Effects of sirolimus treatment on patients with β-thalassemia: Lymphocyte immunophenotype and biological activity of memory CD4+ and CD8+ T cells. J Cell Mol Med. (2023) 27:353–64. doi: 10.1111/jcmm.17655 36625233 PMC9889681

[186] Ju WT Ma HL Zhao TC Liang SY Zhu DW Wang LZ . Stathmin guides personalized therapy in oral squamous cell carcinoma. Cancer Sci. (2020) 111:1303–13. doi: 10.1111/cas.14323 31994271 PMC7156844

[187] Svatek RS Ji N de Leon E Mukherjee NZ Kabra A Hurez V . Rapamycin prevents surgery-induced immune dysfunction in patients with bladder cancer. Cancer Immunol Res. (2019) 7:466–75. doi: 10.1158/2326-6066.CIR-18-0336 30563829 PMC6926429

[188] McCormack FX Inoue Y Moss J Singer LG Strange C Nakata K . Efficacy and safety of sirolimus in lymphangioleiomyomatosis. N Engl J Med. (2011) 364:1595–606. doi: 10.1056/NEJMoa1100391 21410393 PMC3118601

[189] Toniato de Rezende Freschi J Cristelli MP Viana LA Ficher KN Nakamura MR Proença H . A head-to-head comparison of de novo sirolimus or everolimus plus reduced-dose tacrolimus in kidney transplant recipients: A prospective and randomized trial. Transplantation. (2024) 108:261–75. doi: 10.1097/TP.0000000000004749 37525373

[190] Hudes G Carducci M Tomczak P Dutcher J Figlin R Kapoor A . Temsirolimus, interferon alfa, or both for advanced renal-cell carcinoma. N Engl J Med. (2007) 356:2271–81. doi: 10.1056/NEJMoa066838 17538086

[191] Cockerell I Christensen J Hoei-Hansen CE Holst L Grenaa Frederiksen M Issa-Epe AI . Effectiveness and safety of everolimus treatment in patients with tuberous sclerosis complex in real-world clinical practice. Orphanet J Rare Dis. (2023) 18:377. doi: 10.1186/s13023-023-02982-1 38042867 PMC10693167

[192] Yao JC Shah MH Ito T Bohas CL Wolin EM Van Cutsem E . Everolimus for advanced pancreatic neuroendocrine tumors. N Engl J Med. (2011) 364:514–23. doi: 10.1056/NEJMoa1009290 21306238 PMC4208619

[193] Cillo U Saracino L Vitale A Bertacco A Salizzoni M Lupo F . Very early introduction of everolimus in de novo liver transplantation: Results of a multicenter, prospective, randomized trial. Liver Transpl. (2019) 25:242–51. doi: 10.1002/lt.25400 30592371

[194] Wagner AJ Ravi V Riedel RF Ganjoo K Van Tine BA Chugh R . nab-Sirolimus for patients with Malignant perivascular epithelioid cell tumors. J Clin Oncol. (2021) 39:3660–70. doi: 10.1200/JCO.21.01728 34637337 PMC8601264

[195] Lashgari NA Roudsari NM Momtaz S Ghanaatian N Kohansal P Farzaei MH . Targeting mammalian target of rapamycin: Prospects for the treatment of inflammatory bowel diseases. Curr Med Chem. (2021) 28:1605–24. doi: 10.2174/0929867327666200504081503 32364064

